# Heart-liver co-management: epidemiology, mechanisms, and novel therapeutic approaches for metabolic dysfunction-associated steatotic liver disease and coronary artery disease

**DOI:** 10.3389/fcvm.2026.1843719

**Published:** 2026-07-27

**Authors:** Sirui Zhu, Fangkun Yang, Haoxuan Lu, Jun Jiang, Wenming He

**Affiliations:** 1 Department of Cardiology, The Second Affiliated Hospital of Zhejiang University School of Medicine, Zhejiang University, Hangzhou, China; 2 Department of Cardiology, The First Affiliated Hospital of Ningbo University, Ningbo, China

**Keywords:** cardiovascular risk, coronary artery disease, heart-liver co-management, metabolic dysfunction-associated fatty liver disease, treatment

## Abstract

**Background:**

Metabolic dysfunction-associated steatotic liver disease (MASLD) and coronary artery disease (CAD) are interconnected global health epidemics that substantially contribute to worldwide morbidity and mortality. We aimed to provide a systematic overview of the epidemiological links and shared pathophysiological mechanisms between MASLD and CAD, evaluate emerging therapeutic strategies with dual benefits, and advocate for an integrated “dual-heart-liver” management model.

**Methods:**

We synthesize current evidence to examine the epidemiological link between MASLD and CAD and their shared pathophysiological mechanisms, including endothelial dysfunction, chronic inflammation, metabolic imbalance, insulin resistance, and genetic associations. We also discuss the roles of genetic predisposition, lifestyle modification, and combination therapies in improving patient outcomes. Furthermore, recent advances in pharmacotherapy—such as resmetirom, GLP-1 receptor agonists, SGLT2 inhibitors, FGF21 analogues, PCSK9 inhibitors, and PPAR agonists—are evaluated for their dual benefits on hepatic and cardiovascular health

**Results:**

Epidemiological studies confirm a bidirectional, mutually reinforcing relationship between MASLD and CAD, which are linked through multiple pathophysiological pathways. In terms of treatment, novel pharmacotherapies—such as resmetirom, GLP-1 receptor agonists, SGLT2 inhibitors, FGF21 analogues, PCSK9 inhibitors, and PPAR agonists—show considerable potential for conferring concurrent hepatic and cardiovascular benefits. Furthermore, combination therapies also play important roles in optimizing patient outcomes.

**Conclusions:**

This review calls for a paradigm shift toward an integrated “dual-heart-liver” management model, emphasizing the need for systematic cardiovascular risk assessment in MASLD patients and the implementation of collaborative care strategies to improve long-term prognosis.

## 1 Introduction

Metabolic dysfunction-associated fatty liver disease (MASLD), formerly known as non-alcoholic fatty liver disease (NAFLD) or metabolic dysfunction-associated fatty liver disease (MAFLD) (1, 2), and coronary artery disease (CAD) represent two major global public health challenges (1, 3–5). Epidemiological studies establish MASLD as a principal cause of liver-related morbidity and mortality, whereas CAD and its complications rank among the leading causes of adult death worldwide (1, 3–5). Extensive evidence has solidified MASLD as an independent risk factor for atherosclerotic cardiovascular diseases. This association is underscored by the observation that 44.6% of MASLD patients develop CAD, which has emerged as the predominant cause of mortality within this patient population (6, 7). More recent evidence reveals a dose-response relationship between the severity of hepatic steatosis in MASLD and the extent of coronary atherosclerosis (8–10).

This dual burden should not be regarded as a simple coexistence of diseases, but as a complex interplay driven by mechanisms such as metabolic imbalance, genetic background, chronic inflammation, and immune response (11, 12). In recent years, clinical medicine has achieved breakthrough in both fields. For MASLD, novel treatments targeting metabolic and inflammatory pathways have emerged, including resmetirom (13–15), glucagon-like peptide-1 receptor agonists (GLP-1RAs) (16, 17), sodium-glucose cotransporter 2 inhibitors (SGLT2i) (18, 19) and fibroblast growth factor 21 analogs (FGF21 analogs) (20–22). Concurrently, the cardiovascular field has increasingly recognized the cardioprotective benefits of therapies extending beyond lipid-lowering (23, 24). Systematically evaluating the dual benefits and translational potential of these drugs for the heart-liver axis represents a central challenge in current research.

This article systematically reviews the established epidemiological association between MASLD and CAD, their shared pathological mechanisms, and the dual effects of emerging pharmacological agents. These insights inform future clinical treatment strategies and trial designs, supporting the practical implementation of integrated heart-liver management.

## 2 The epidemiological association between MASLD and CAD

Metabolic dysfunction-associated steatotic liver disease (MASLD), formerly referred to as non-alcoholic fatty liver disease (NAFLD), is currently defined as hepatic steatosis occurring in the presence of at least one cardiometabolic risk factor and in the absence of harmful alcohol intake or other identifiable causes of steatotic liver disease (1, 25). Its disease spectrum ranges from simple steatosis to metabolic dysfunction-associated steatohepatitis, progressive fibrosis, cirrhosis, and liver-related complications (25). Coronary artery disease (CAD) is a major manifestation of atherosclerotic cardiovascular disease and is characterized by atherosclerotic involvement of the coronary arteries, which may lead to myocardial ischemia and clinically present as chronic coronary disease, prior myocardial infarction, previous coronary revascularization, or acute coronary syndromes (26, 27). Because cardiometabolic dysfunction, insulin resistance, dyslipidemia, systemic inflammation, and endothelial injury are involved in both MASLD and CAD, the coexistence of these two diseases may not be accidental. Instead, they may share common metabolic and vascular mechanisms.

Since MASLD is a newly adopted term, most available data in CAD populations come from studies that used the older term NAFLD. Despite this difference in terminology, these studies provide useful evidence supporting the association between fatty liver disease and CAD or ACS (1).

Table 1 summarizes the epidemiological evidence linking MASLD with CAD. Figure 2 shows a dual-pathway risk diagram epidemiological association and prognostic impact of MAFLD on CAD.

**Table 1:** Summary of studies that evaluated the association between NAFLD and clinical CAD.

Section	Study/source	Study type/population	Key epidemiological findings related to MASLD/NAFLD and CAD	Interpretation
2.1 Impact of MASLD on CAD risk	Ren et al. 2023 (28)	Mendelian randomization study	Evaluated the relationship between genetically predicted NAFLD and CAD.	Provides genetic epidemiological evidence supporting a NAFLD–CAD relationship.
2.1 Impact of MASLD on CAD risk	Simon et al. 2023 (29)	Pediatric and young adult NAFLD cohort	NAFLD was associated with increased cardiovascular disease risk in children and young adults.	Suggests that cardiovascular risk may emerge early in the course of fatty liver disease.
2.1 Impact of MASLD on CAD risk	Alon et al. 2022 (30)	Systematic review and meta-analysis	NAFLD was associated with higher risks of cardiovascular events, including myocardial infarction and ischemic stroke.	Supports NAFLD as a clinically relevant cardiovascular risk factor.
2.1 Impact of MASLD on CAD risk	Clayton-Chubb et al. 2025 (31)	Older adult cohort study	MASLD was associated with atrial fibrillation, cardiovascular events, mortality, and aspirin-related outcomes in older adults.	Extends the cardiovascular relevance of MASLD to aging populations.
2.1/2.3	Mantovani et al. 2021 (32)	Updated systematic review and meta-analysis	NAFLD increased the risk of fatal or non-fatal cardiovascular disease by approximately 45%; advanced fibrosis was associated with a markedly higher cardiovascular risk.	Supports a severity-dependent relationship between fatty liver disease and cardiovascular events.
2.1 Impact of MASLD on CAD risk	Toh et al. 2022 (6)	Meta-analysis	CAD/CHD was common in NAFLD, and diabetes, hypertension, and age were major risk factors for CAD in this population.	Supports cardiovascular risk-factor screening in NAFLD/MASLD patients.
2.1 Impact of MASLD on CAD risk	Au Yeung et al. 2023 (33)	Mendelian randomization study in Europeans and East Asians	The NAFLD–CAD association showed ethnic heterogeneity, with a positive association in Europeans and an inverse association in East Asians.	Highlights the need for population-specific cardiovascular risk assessment in MASLD/NAFLD.
2.1 Impact of MASLD on CAD risk	Flessa et al. 2022 (34)	Review of genetic and diet-induced NAFLD models	Summarized genetic and dietary factors involved in NAFLD pathogenesis.	Supports the interpretation that genetic and dietary exposures may modify MASLD-related CAD risk across populations.
2.1 Impact of MASLD on CAD risk	Lindén and Romeo, 2023 (35)	Review of genetically validated NASH targets	Discussed genetically supported targets involved in NASH biology, including lipid-handling pathways.	Supports the role of genetic susceptibility in fatty liver phenotypes.
2.1 Impact of MASLD on CAD risk	Lee et al. 2024 (36)	Experimental genetic/metabolic study	Investigated SREBP-1c-related hepatic lipid and fibrosis pathways.	Supports discussion of lipid-metabolism-related genetic mechanisms in MASLD.
2.1 Impact of MASLD on CAD risk	Nisar et al. 2023 (37)	Case-control genetic study	Examined the GCKR rs1260326 variant in obesity-associated NAFLD and T2DM.	Supports population-specific genetic heterogeneity in metabolic fatty liver disease.
2.1 Impact of MASLD on CAD risk	Gobeil et al. 2024 (38)	Genetic cardiometabolic study	Linked genetically influenced lipid pathways with cardiometabolic risk.	Supports the connection between lipid-related genetic pathways and cardiovascular risk.
2.1 Impact of MASLD on CAD risk	Brouwers et al. 2020 (39)	Review assessing causality between NAFLD and CVD	Discussed whether the NAFLD–CVD association is causal or confounded by shared metabolic risk factors.	Provides context for interpreting the epidemiological association between NAFLD and CAD/CVD.
2.1 Impact of MASLD on CAD risk	Saki et al. 2020 (40)	Review of genetic aspects of fatty liver and premature cardiovascular events	Summarized genetic polymorphisms related to fatty liver disease and premature cardiovascular events.	Supports the role of genetic susceptibility in NAFLD-related cardiovascular risk.
2.1 Impact of MASLD on CAD risk	Gariani and Jornayvaz, 2021 (41)	Review of NASH pathophysiology in endocrine disease	Discussed diet, insulin resistance, obesity, dyslipidemia, T2DM, metabolic syndrome, and endocrine factors in NASH.	Supports the role of dietary and metabolic factors in MASLD–CAD overlap.
2.1 Impact of MASLD on CAD risk	Lee et al. 2018 (42)	Observational cohort of 5,121 asymptomatic Korean adults	NAFLD was independently associated with a higher prevalence of non-calcified coronary plaques; the association persisted after adjustment for cardiovascular risk factors.	Suggests that NAFLD may be linked to early and potentially vulnerable coronary atherosclerosis.
2.1/2.3	Simon et al. 2022 (43)	Nationwide histology cohort	Biopsy-confirmed NAFLD was associated with incident MACE, including ischemic heart disease, heart failure, stroke, and cardiovascular death.	Links histologically confirmed NAFLD with future cardiovascular events and prognosis.
2.1 Impact of MASLD on CAD risk	Carter et al. 2022 (44)	Coronary CT angiography study	Hepatosteatosis was associated with coronary atherosclerotic plaque characteristics.	Suggests that liver fat may be related to coronary plaque burden and plaque phenotype.
2.1 Impact of MASLD on CAD risk	Ichikawa et al. 2024 (45)	Multi-Ethnic Study of Atherosclerosis	Liver fat accumulation was associated with earlier progression from CAC score 0 to detectable coronary calcification.	Indicates that hepatic steatosis may shorten the low-risk period after CAC score 0 and promote early coronary calcification.
2.1 Impact of MASLD on CAD risk	Park et al. 2016 (46)	Longitudinal cohort study	NAFLD was associated with new-onset coronary artery calcification, especially among individuals with baseline CAC score 0.	Suggests that NAFLD may contribute mainly to the early phase of coronary calcification development.
2.2 Prevalence of MASLD in the CAD population	Friedrich-Rust et al. 2017 (47)	Prospective coronary angiography cohort	NAFLD was highly prevalent and more frequent among patients with severe CAD.	Supports an association between fatty liver disease and angiographic CAD severity.
2.2 Prevalence of MASLD in the CAD population	Boddi et al. 2013 (48)	Nondiabetic ACS cohort	NAFLD was common in nondiabetic ACS patients and was associated with more severe coronary involvement and adverse cardiovascular outcomes.	Indicates that the NAFLD–CAD association is not restricted to diabetic patients.
2.2/2.3	Wong et al. 2016 (49)	Prospective cohort of patients undergoing coronary angiography	NAFLD was associated with coronary artery stenosis and a higher likelihood of PCI, although its independent association with long-term outcomes was less consistent after adjustment.	Suggests that NAFLD reflects CAD burden, but its independent prognostic value may vary.
2.2 Prevalence of MASLD in the CAD population	Agaç et al. 2013 (50)	Pilot study in ACS patients	NAFLD was detected in 81.2% of ACS patients and was associated with higher SYNTAX scores.	Links NAFLD with greater coronary lesion complexity.
2.2 Prevalence of MASLD in the CAD population	Baharvand-Ahmadi et al. 2016 (51)	Prospective study of angiographically confirmed CAD patients	NAFLD was present in 47% of patients with angiographically confirmed CAD.	Demonstrates a substantial overlap between fatty liver disease and established CAD.
2.2 Prevalence of MASLD in the CAD population	Montemezzo et al. 2020 (10)	Prospective study in ACS patients	NAFLD was present in 55.2% of ACS patients; among patients with grade III NAFLD, 83.3% had severe CAD.	Suggests that NAFLD severity may help identify ACS patients with more advanced coronary disease.
2.2 Prevalence of MASLD in the CAD population	Wong et al. 2011 (52)	Clinical cohort study	NAFLD was associated with CAD and cardiovascular outcomes, with prognostic effects influenced by metabolic and coronary risk context.	Indicates that fatty liver disease should be interpreted together with cardiometabolic risk factors and CAD severity.
2.2 Prevalence of MASLD in the CAD population	Noda et al. 2022 (53)	Hospitalized ACS cohort using MAFLD definition	MAFLD was common in ACS patients and was associated with impaired physical function and worse prognosis.	Supports the clinical relevance of metabolic dysfunction-based fatty liver definitions in ACS.
2.2/2.3	Liu et al. 2021 (54)	Matched case-control study in chronic coronary syndrome	MAFLD was significantly associated with a higher risk of future MACE in patients with chronic coronary syndrome.	Suggests that metabolic dysfunction-associated fatty liver disease may worsen prognosis in established coronary disease.
2.2 Prevalence of MASLD in the CAD population	Bhardwaj and Sharma, 2025 (55)	Angiography-based CAD study	NAFLD was present in 60.4% of the overall cohort and 77.03% of patients with significant CAD; higher NAFLD grade corresponded to more affected coronary vessels.	Supports a grade-dependent association between NAFLD and CAD extent.
2.3 Prognosis of MASLD patients is closely linked to CAD	Vilar-Gomez et al. 2018 (56)	Multinational cohort of advanced NAFLD	Fibrosis severity was a determinant of cause-specific mortality in advanced NAFLD.	Emphasizes fibrosis stage as a major prognostic marker in NAFLD.
2.3 Prognosis of MASLD patients is closely linked to CAD	Hagström et al. 2017 (57)	Biopsy-proven NAFLD cohort	Fibrosis stage, rather than NASH itself, predicted mortality and severe liver disease.	Suggests that fibrosis should be prioritized in long-term risk stratification.
2.3 Prognosis of MASLD patients is closely linked to CAD	Taylor et al. 2020 (58)	Systematic review and meta-analysis	Higher fibrosis stages were associated with worse all-cause and liver-related outcomes.	Confirms the prognostic importance of fibrosis stage.
2.3 Prognosis of MASLD patients is closely linked to CAD	Dulai et al. 2017 (59)	Systematic review and meta-analysis	Mortality risk increased progressively with advancing fibrosis stage in NAFLD.	Reinforces fibrosis stage as a major determinant of long-term prognosis.
2.3 Prognosis of MASLD patients is closely linked to CAD	Angulo et al. 2015 (60)	Multicenter biopsy-proven NAFLD cohort	Liver fibrosis, but not other histologic features, was associated with long-term outcomes.	Supports fibrosis burden as a central prognostic marker in NAFLD.
2.3 Prognosis of MASLD patients is closely linked to CAD	Duell et al. 2022 (7)	American Heart Association scientific statement	Recognized NAFLD as a risk factor for atherosclerotic cardiovascular disease and emphasized cardiovascular risk assessment.	Provides authoritative support for routine cardiovascular risk assessment in NAFLD/MASLD.
2.3 Prognosis of MASLD patients is closely linked to CAD	Targher et al. 2016 (61)	Meta-analysis	NAFLD was associated with increased incident cardiovascular disease.	Supports the long-term cardiovascular risk of NAFLD.
2.3 Prognosis of MASLD patients is closely linked to CAD	Moon et al. 2023 (62)	Nationwide cohort study using MASLD definition	MASLD was associated with a higher risk of incident cardiovascular disease.	Provides updated epidemiological evidence under the MASLD definition.
2.3 Prognosis of MASLD patients is closely linked to CAD	Lee et al. 2024 (63)	Population-based cohort study using MASLD definition	MASLD was associated with increased future cardiovascular disease risk.	Further supports the prognostic relevance of MASLD under the new nomenclature.
2.3 Prognosis of MASLD patients is closely linked to CAD	Shi et al. 2024 (64)	Coronary CT angiography cohort	Moderate-to-severe NAFLD independently predicted MACE, especially in patients with obstructive CAD.	Suggests that NAFLD severity may improve prognostic assessment in patients evaluated by CCTA.

### 2.1 The impact of MASLD on CAD risk

CAD falls under the category of cardiovascular diseases (CVD), which encompasses all disorders of the heart and blood vessels. Given that CAD is the most prevalent form of CVD, numerous investigations into the general relationship between MASLD and CVD have substantially affirmed the close connection between CAD and MASLD to a large extent.

Multiple cohort studies and meta-analyses have established MASLD as an independent risk factor for CAD (28–31). Compared with individuals without MASLD, patients with MASLD appear to be at higher risk of both fatal and non-fatal cardiovascular events, including myocardial infarction, ischemic stroke, atrial fibrillation, and heart failure. The odds ratios were 1.66, 1.41, 1.27, and 1.62 respectively (30, 31). Furthermore, a longitudinal study involving 5.8 million middle-aged and elderly individuals found that NAFLD increases the risk of fatal or non-fatal cardiovascular disease by 45% (HR 1.45), with the risk significantly rising with the severity of NAFLD (32).What's more, the severity of MASLD is positively correlated with the incidence of CAD (6). In the liver fibrosis stage, the cardiovascular risk increase reaches 150% (HR 2.50) (32). Additionally, diabetes, hypertension, and age were identified as the main risk factors for the development of CAD in these patients (6). The association between NAFLD and CAD risk demonstrates ethnic heterogeneity, with a positive correlation observed in European populations (OR = 1.27) but an inverse association found in East Asian groups (OR = 0.63) (33). The observed ethnic heterogeneity in the MASLD–CAD association may reflect differences in genetic susceptibility, dietary exposure, and population-specific metabolic phenotypes. Variants involved in hepatic lipid handling and cardiometabolic regulation, including PNPLA3, TM6SF2, GCKR, MBOAT7, HSD17B13, and APOC3, may differ in frequency and biological impact across populations (33–40). In addition, regional dietary patterns, such as high intake of refined carbohydrates, fructose, saturated fat, or total calories, may influence hepatic *de novo* lipogenesis, insulin resistance, and atherogenic dyslipidemia (41). Body-fat distribution and metabolic profiles may also vary by ethnicity. Some Asian populations develop MASLD and cardiometabolic complications at lower body mass index levels than Western populations. These factors may partly explain why the MASLD–CAD association differs across ethnic groups. Future multi-ethnic studies are needed to determine whether CAD risk prediction in MASLD should be adjusted according to population-specific genetic, dietary, and metabolic characteristics.

In terms of atherosclerotic phenotypes, research has revealed how NAFLD specifically affects different types of plaques. NAFLD may be an independent risk factor for non-calcified plaques (42). A cross-sectional study of 5,121 asymptomatic Korean adults revealed a significantly higher prevalence of non-calcified plaques in individuals with NAFLD than in those without. NAFLD persisted as an independent risk factor for non-calcified plaques even after adjustment for common cardiovascular risk factors (OR=1.27) (42).Another study found that NAFLD patients had a higher occurrence of both non-calcified and mixed plaques, which are considered unstable and have a greater risk of rupture (43, 44). These vulnerable plaques are closely tied to the risk of acute coronary syndrome, further highlighting the crucial role NAFLD plays in the progression of cardiovascular events.

NAFLD has been established as an independent risk factor for the development of coronary artery calcified plaques and the progression of coronary artery calcification (CAC) (45). A longitudinal cohort study found that among those with a baseline coronary artery calcium (CAC) score of 0, NAFLD patients had a significantly higher risk of developing new calcifications (OR=1.49). However, in individuals with existing CAC at baseline, NAFLD did not significantly accelerate the progression of calcification, with diabetes emerging as the main driving factor at this stage (46). Furthermore, another study indicates that 25% of those with liver fat accumulation experience calcification much earlier than those without, with the time to calcification being notably shorter (4.7 years vs. 6.3 years) (45). These findings indicate that NAFLD contributes primarily to the early phase of coronary atherosclerosis and represents an independent risk factor for the initial development of coronary artery calcification. This progression likely involves underlying mechanisms such as systemic inflammation, insulin resistance, and metabolic dysfunction (45).

Despite incomplete adjustment for confounders, studies consistently demonstrate a strong association between MASLD and elevated CAD risk. This association establishes a clinical necessity for early screening and intervention in this population.

### 2.2 Prevalence of MASLD in the CAD population

Overall, existing data consistently indicate that fatty liver disease is highly prevalent among patients with angiographically confirmed CAD or ACS, with reported prevalence ranging from approximately 47% to more than 80%, depending on the study population, diagnostic modality, and definition of CAD severity (47–51). In a prospective study of 170 patients with angiographically confirmed CAD, concurrent NAFLD was observed in 47% of cases; however, NAFLD was not significantly associated with sex, diabetes, hyperlipidemia, hypertension, or left anterior descending artery involvement (51). Similarly, Montemezzo et al. reported that 55.2% of patients with ACS had NAFLD, and that NAFLD severity was closely related to the degree of coronary artery obstruction. Notably, among patients with grade III NAFLD, 83.3% had severe CAD, suggesting that fatty liver severity may help identify ACS patients with more advanced coronary atherosclerosis (10).

This severity-dependent relationship has been further supported by angiography-based studies. Agaç et al. found that NAFLD was present in 81.2% of ACS patients and was associated with higher SYNTAX scores, indicating greater coronary lesion complexity (50). In nondiabetic patients with ACS, Boddi et al. also reported a high prevalence of NAFLD and suggested that more severe hepatic steatosis was associated with multivessel coronary involvement and adverse cardiovascular outcomes (48) Consistently, Friedrich-Rust et al. showed in a single-blinded prospective cohort of patients undergoing coronary angiography that NAFLD was highly prevalent and more frequent among those with severe CAD (47). Wong et al. further demonstrated that, among patients undergoing coronary angiography, NAFLD was associated with coronary artery stenosis and a higher likelihood of requiring percutaneous coronary intervention, although its relationship with long-term mortality or composite cardiovascular outcomes was less consistent (49) Earlier angiography-based evidence also supported an association between NAFLD and CAD, but highlighted that the prognostic implications of fatty liver in CAD populations may depend on metabolic comorbidities, CAD severity, and follow-up duration (52).

More recent studies using metabolic dysfunction–based definitions provide additional support for this concept. Noda et al. reported that metabolic dysfunction-associated fatty liver disease was common among hospitalized patients with ACS and was associated with impaired physical function and worse prognosis (53). In patients with chronic coronary syndrome, Liu et al. showed that metabolic-associated fatty liver disease was significantly associated with a higher risk of major adverse cardiac events during follow-up (54). A recent angiography-based study also reported that NAFLD was present in 60.4% of the overall cohort and in 77.03% of patients with significant CAD, with higher NAFLD grades corresponding to a greater number of affected coronary vessels (55).

Taken together, these findings suggest that MASLD/NAFLD is not merely common in CAD and ACS populations, but may also reflect coronary atherosclerotic burden, lesion complexity, and future cardiovascular risk. However, the reported prevalence differs considerably across studies, reflecting variations in diagnostic methods, ethnicity, cardiometabolic profiles, and criteria used to define CAD severity. In addition, many studies were limited by single-center design and small sample size. Thus, while MASLD/NAFLD assessment may help improve cardiovascular risk stratification when interpreted alongside traditional risk factors and angiographic findings, larger multicenter prospective studies are needed to clarify its clinical utility.

### 2.3 The prognosis of MASLD patients is closely linked to CAD

Longitudinal cohort studies and meta-analyses indicate that the prognosis of patients with MASLD/NAFLD is primarily driven by liver fibrosis rather than steatosis alone. Higher fibrosis stage is consistently associated with increased all-cause mortality, liver-related mortality, and severe liver outcomes, whereas the presence of steatohepatitis itself appears less predictive after accounting for fibrosis (7, 56–60). However, the long-term burden of MASLD extends beyond liver failure or hepatocellular carcinoma. Cardiovascular disease, particularly CAD-related events, represents a leading cause of non-liver morbidity and mortality in this population (7, 32, 43, 61). In a nationwide histology cohort, patients with biopsy-confirmed NAFLD showed a higher incidence of major adverse cardiovascular events (MACE), including ischemic heart disease, heart failure, stroke, and cardiovascular death, with this risk increasing alongside more advanced liver disease (43). More recent studies using the MASLD definition have also confirmed a higher risk of incident cardiovascular disease (62, 63).

Evidence from CAD-focused cohorts further supports this link. Among patients with chronic coronary syndrome, MAFLD was associated with a higher risk of MACE (54). Similarly, in patients evaluated by coronary computed tomography angiography, moderate-to-severe NAFLD independently predicted MACE, particularly in those with obstructive CAD (64). However, not all angiography-based studies have demonstrated a clear independent association between fatty liver and long-term cardiovascular outcomes after adjusting for established coronary disease (49).

## 3 Heart–liver crosstalk mechanism

### 3.1 Endothelial dysfunction and vascular structural changes

Endothelial dysfunction occurring during the progression of MASLD is considered an independent risk factor for the development of CAD (65). Endothelial dysfunction represents an early vascular lesion in atherosclerotic patients, preceding the formation of fatty streaks or plaque inflammation, and plays a crucial role in the onset and progression of CAD (66, 67). Impaired endothelial function is a prominent feature in patients with MASLD, especially in those with MASH (68). Nitric oxide (NO) is an endothelium-derived vasodilator, whose synthesis is negatively regulated by asymmetric dimethylarginine (ADMA), an endogenous antagonist of nitric oxide synthase (NOS) (69, 70). In patients with NAFLD, impaired hepatic clearance of ADMA leads to elevated circulating levels, which in turn suppresses NO production—a mechanism closely associated with the severity of CVD (71).

An intact endothelial monolayer is essential for normal vascular wall function, and the disruption of endothelial structural integrity is a characteristic feature in patients with CAD (72). This is reflected by increased levels of endothelial microparticles (EMPs), indicating endothelial injury (73, 74), and a reduced level of endothelial progenitor cells (EPCs), which are crucial for endothelial repair, as observed in patients with MASLD (74, 75). Furthermore, multiple factors associated with vascular dysfunction are altered in MASLD. Elevated levels of Endocan are linked to endothelial dysfunction in MASLD patients and correlate with the severity of endothelial impairment in CAD (76). Increased soluble CD40 Ligand (sCD40L) accelerates endothelial activation, inflammation (77) and vascular injury while increased Endothelin-1 (ET-1) inhibits vasodilation and promotes inflammatory responses (78). Conversely, decreased levels of protective factors further contribute to endothelial dysfunction. These include declines in HBGM-1, which helps maintain endothelial function; adiponectin, which exerts anti-inflammatory and anti-atherosclerotic effects; and angiopoietin-2 (Ang-2), involved in angiogenesis and vascular stability (79, 80). Additionally, several studies have confirmed that leptin, the renin-angiotensin system (RAS), and C-reactive protein (CRP) are closely associated with endothelial dysfunction (81–83).

### 3.2 Insulin resistance

The accumulation of intrahepatic steatosis is associated with the buildup of intrahepatic ceramides and diacylglycerols, which have been shown to inhibit insulin signaling, thereby leading to hepatic insulin resistance (84). Studies indicate that patients with MASLD may experience impaired function of endothelial nitric oxide synthase (eNOS) due to insulin resistance, resulting in reduced production of nitric oxide (NO) and an imbalance in platelet-mediated induction of vasodilation (69).

Insulin resistance is a key mechanism in the development of T2DM (85). An epidemiological Mendelian randomization study reported that genetic susceptibility to hyperglycemia is associated with an increased risk of CAD, demonstrating a causal effect and indirectly illustrating the role of insulin resistance in promoting CAD (86). Studies have demonstrated that postprandial hyperlipidemia induced by insulin resistance may contribute to coronary artery disease by promoting the adhesion of monocytes/macrophages to the endothelium, enhancing the proliferation of vascular smooth muscle cells, or directly impacting the arterial wall structure through induced endothelial dysfunction and inflammatory macrophages (87–89).

Recent studies demonstrate significant spatial heterogeneity in hepatic insulin signaling, which plays distinct roles in metabolic regulation. Insulin resistance in specific liver regions can even ameliorate hepatic steatosis, challenging the conventional view that it uniformly promotes lipid deposition (90). Consequently, the precise mechanisms by which insulin resistance influences both NAFLD and CAD require further elucidation through more refined animal models and larger clinical cohorts.

### 3.3 Lipotoxicity

The liver serves as a central regulator of systemic lipid metabolism, functioning through *de novo* lipogenesis, lipid breakdown, and the uptake and secretion of serum lipoproteins (91). In patients with NAFLD, there is an increased proportion of small dense LDL and very-low-density lipoprotein (VLDL), a high apolipoprotein B and apolipoprotein A-1 ratio, and low high-density lipoprotein (HDL) cholesterol concentrations (92, 93). Dyslipidemia is a common risk factor for atherosclerosis; increased levels of triglycerides (TG) and LDL, coupled with reduced HDL, lead to a more atherogenic lipid profile, which contributes to postprandial hyperlipidemia in NAFLD patients. This condition further progresses to accelerated postprandial atherogenesis and a higher risk of CAD (94, 95).

Compared to NAFLD, NASH is associated with more small dense LDL particles (LDL3 and LDL4) and less large LDL particles (LDL1) (96). Small dense LDL particles are considered a significant risk factor for CAD (97). The NAFLD-CVD link is also influenced by adipokine-driven modifications in the lipid profile, involving adiponectin, fibroblast growth factor-21 (FGF-21) (98), and fatty acid-binding protein (FABP) (99). Low adiponectin levels in NAFLD patients, particularly those with more severe disease, contribute to an increased CAD risk (81). FGF-21, which may improve hepatic steatosis and fibrosis and has potential vasculoprotective effects, is compensatively elevated in NAFLD patients. Moreover, recent studies on FGF-21 analogue drugs suggest dual protective benefits for both cardiovascular disease and NAFLD patients (98, 100). FABP, a key mediator of macrophage inflammatory responses (99), promotes foam cell formation in CAD patients and is expressed at higher levels in NASH patients compared to non-NASH individuals (101).

### 3.4 Chronic inflammation and immune activation

Elevated inflammatory status and oxidative stress play a critical role in coronary artery disease associated with NAFLD. The stability of atherosclerotic plaques is directly compromised by the systemic inflammation linked to NAFLD (76). During the progressive stage of MASLD, particularly in MASH, hepatic lipid overload and lipotoxicity elevate ROS production through mitochondrial dysfunction, endoplasmic reticulum stress, NADPH oxidase activation, and excessive fatty acid oxidation (102–105). Elevated ROS drive lipid peroxidation, oxidative DNA damage, and hepatocyte injury, thereby activating inflammatory pathways such as JNK, NF-*κ*B, and inflammasome signaling. In this manner, oxidative stress contributes to both liver inflammation and the transition from simple steatosis to MASH.

Oxidative stress, however, is not confined to the liver. Oxidized lipids and lipid peroxidation products can enter the circulation and act as danger signals, linking hepatic injury to vascular inflammation (106). In the vascular wall, ROS reduce nitric oxide bioavailability, aggravate endothelial dysfunction, and promote LDL oxidation. Oxidized LDL and oxidized phospholipids then enhance monocyte recruitment, macrophage foam cell formation, and inflammatory cell activation, all of which accelerate atherosclerotic plaque formation and progression (105–108). Oxidative stress may also weaken plaque stability by increasing vascular inflammation, smooth muscle cell injury, extracellular matrix degradation, and thrombogenic activity, thereby raising the risk of CAD-related events (106, 107).

During the progressive stage of MASLD—specifically in MASH—the liver also abnormally releases multiple pro-inflammatory and pro-atherogenic mediators. These include: interleukin-6 (IL-6), interleukin-17 (IL-17), interleukin-1β (IL-1β), tumor necrosis factor-α (TNF-α), high-sensitivity C-reactive protein (hs-CRP), leptin, adiponectin, tissue plasminogen activator (t-PA), plasminogen activator inhibitor-1 (PAI-1), monocyte chemoattractant protein-1 (MCP-1), CXCL16, free fatty acids, and other related molecules (109–111). These mediators induce a systemic low-grade inflammatory state and promote the initiation and progression of atherosclerosis by activating inflammatory pathways, recruiting inflammatory cells, impairing endothelial function, and interacting with oxidative stress-related pathways.

### 3.5 Genetic associations

Genetic studies, particularly on variants in genes such as PNPLA3, TM6SF2 (34, 35), SREBP (36), MAT1A, ALMS1 (34), and GCKR (37) indicate that altered lipid metabolism is central to the association between NAFLD and Coronary Artery Disease (CAD) (38, 39, 80). Notably, variants in PNPLA3,TM6SF2 and SREBP appear to reduce CAD risk by lowering plasma lipid levels (36), whereas MAT1A,ALMS1 and GCKR variants increase CAD risk by elevating them (80).

Furthermore, numerous other genetic polymorphisms have been documented in the MASLD-CVD association. The most frequently reported include those in HSD17B13, the adiponectin-encoding gene (ADIPOQ), APOC3, PPAR, LEPR, TNF-α, MTTP, MnSOD, MBOAT7, and the DYRK1B p.Arg102Cys mutation (40).

Since the precise relationship between the genomic signatures of NAFLD and CVD beyond chance associations is not yet fully established, there is a pressing need for large-scale, multi-center cohort studies utilizing advanced genomic assessment and sequencing technologies to clarify the genetic links between NAFLD and CAD.

### 3.6 Shared risk factors

Established determinants of NAFLD progression include unhealthy lifestyles such as physical inactivity, dietary patterns characterized by excessive saturated fat or fructose intake, and *de novo* lipogenesis from high carbohydrate consumption. Additional drivers involve microbial-derived metabolites like fatty acids, as well as metabolic comorbidities including insulin resistance, obesity, dyslipidemia, type 2 diabetes, metabolic syndrome, and hypothyroidism. Other contributing factors comprise obstructive sleep apnea, hypertension, alterations in gut microbiota, and genetic susceptibility (41).

Recognized common risk factors for CAD include age, sex, family history of premature CVD, hypertension, hyperlipidemia, obesity, T2DM, chronic smoking, and other comorbidities that elevate CVD risk (112).

### 3.7 Crosstalk among shared mechanisms linking MASLD and CAD

**Figure 1:**
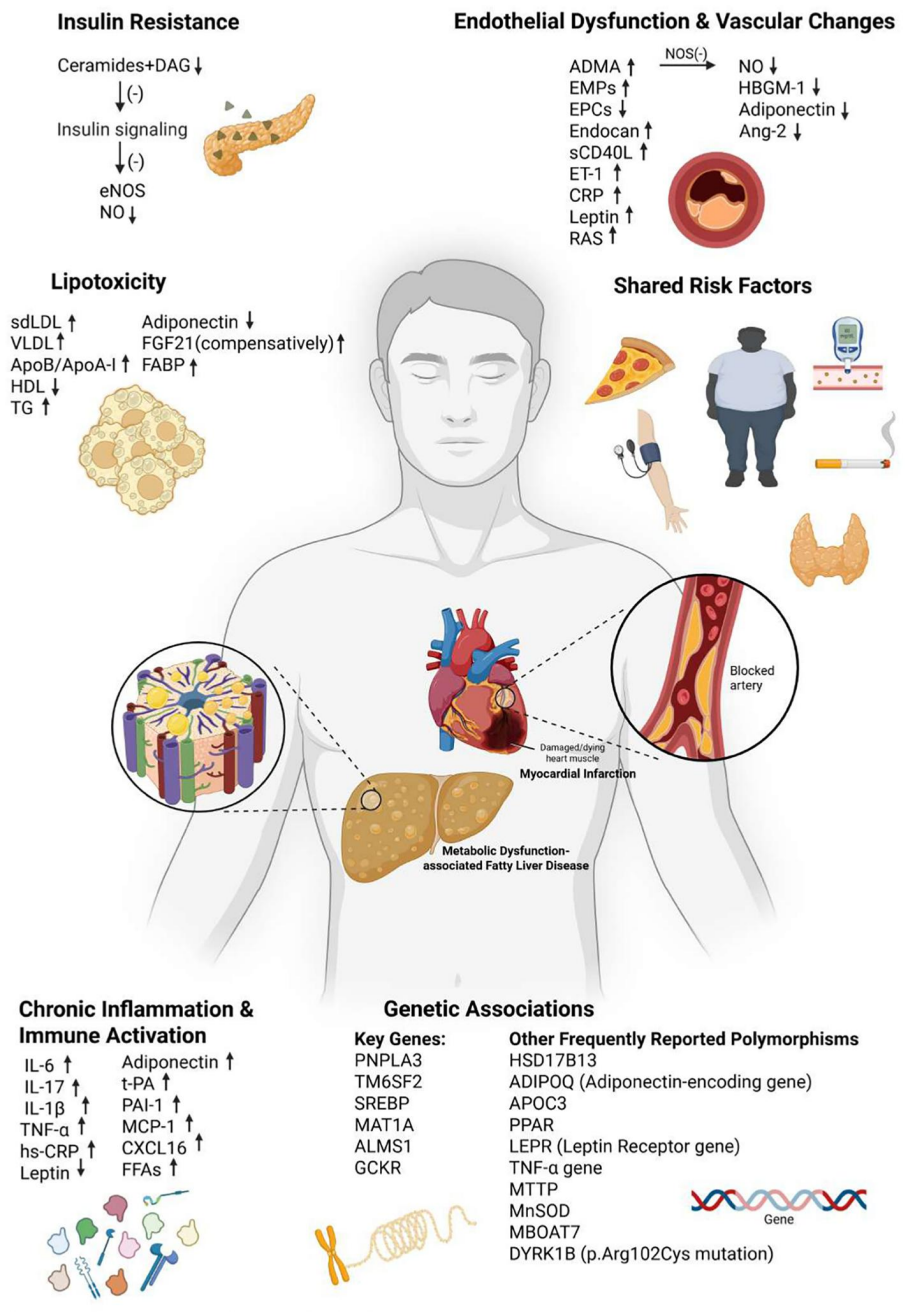
Summary of suggested pathophysiological mechanisms of the NAFLD–CAD interrelationship. This schematic summarizes the major biological pathways linking metabolic dysfunction-associated fatty liver disease (MAFLD) with cardiovascular injury, atherosclerosis, and myocardial infarction. MAFLD is associated with insulin resistance, lipotoxicity, chronic inflammation and immune activation, endothelial dysfunction, vascular remodeling, shared cardiometabolic risk factors, and genetic susceptibility. Upward and downward arrows indicate reported increases or decreases in circulating or tissue biomarkers. Shared risk factors include unhealthy diet, obesity, hypertension, diabetes, smoking, and thyroid dysfunction. These interconnected mechanisms may promote vascular injury, plaque formation, arterial obstruction, and subsequent cardiovascular events. NAFLD, non-alcoholic fatty liver disease; CAD, coronary artery disease; DAG, diacylglycerols; eNOS, endothelial nitric oxide synthase; NO, nitric oxide; sdLDL, small, dense low-density lipoprotein; VLDL, very-low-density lipoprotein; ApoB, apolipoprotein B; ApoA-1, apolipoprotein A-1; HDL, high-density lipoprotein; TG, triglycerides; FGF21, fibroblast growth factor 21; FABP, fatty acid-binding protein; ADMA, asymmetric dimethylarginine; EMPs, endothelial microparticles; EPCs, endothelial progenitor cells; HBGM-1, high mobility group box 1; Ang-2, angiopoietin-2; sCD40L, soluble CD40 ligand; ET-1, endothelin-1; CRP, C-reactive protein; RAS, renin-angiotensin system; IL-6, interleukin-6; IL-17, interleukin-17; IL-1β, interleukin-1 beta; TNF-α, tumor necrosis factor-alpha; hs-CRP, high-sensitivity C-reactive protein; t-PA, tissue plasminogen activator; PAI-1, plasminogen activator inhibitor-1; MCP-1, monocyte chemoattractant protein-1; CXCL16, C-X-C motif chemokine ligand 16; FFAs, free fatty acids; PNPLA3, patatin-like phospholipase domain-containing protein 3; TM6SF2, transmembrane 6 superfamily member 2; SREBP, sterol regulatory element-binding proteins; MAT1A, methionine adenosyltransferase 1A; ALMS1, Alström syndrome protein 1; GCKR, glucokinase regulator; HSD17B13, hydroxysteroid 17-beta dehydrogenase 13; ADIPOQ, adiponectin-encoding gene; APOC3, apolipoprotein C3; PPAR, peroxisome proliferator-activated receptor; LEPR, leptin receptor; MTTP, microsomal triglyceride transfer protein; MnSOD, manganese superoxide dismutase; MBOAT7, membrane bound O-acyltransferase domain containing 7; DYRK1B, dual specificity tyrosine phosphorylation regulated kinase 1B.

Although the shared mechanisms between MASLD and CAD are discussed separately, they are closely connected. In MASLD, hepatic lipid accumulation promotes the buildup of ceramides and diacylglycerols, which impairs insulin signaling and contributes to hepatic insulin resistance (84). Insulin resistance can further reduce endothelial nitric oxide synthase activity and nitric oxide production, while also promoting postprandial hyperlipidemia, monocyte/macrophage adhesion, vascular smooth muscle cell proliferation, and endothelial injury (69, 87–89). At the same time, abnormal hepatic lipid handling leads to an atherogenic lipid profile, characterized by increased VLDL, triglycerides, small dense LDL particles, and reduced HDL cholesterol, thereby enhancing lipotoxicity and coronary atherogenesis (91–97). In progressive MASLD, particularly MASH, lipid overload also increases oxidative stress through mitochondrial dysfunction, endoplasmic reticulum stress, NADPH oxidase activation, and excessive fatty acid oxidation (102–105). Excessive ROS then promotes lipid peroxidation, LDL oxidation, inflammatory cell recruitment, macrophage foam cell formation, and plaque progression (105–108). These oxidative changes interact with liver-derived pro-inflammatory and pro-atherogenic mediators, including IL-6, IL-17, IL-1β, TNF-α, hs-CRP, MCP-1, CXCL16, free fatty acids, and other related molecules, creating a systemic low-grade inflammatory state (109–111). This inflammatory and oxidative environment aggravates endothelial dysfunction, reduces vascular repair capacity, and contributes to coronary plaque instability (74). Genetic susceptibility and shared cardiometabolic risk factors, including obesity, type 2 diabetes, hypertension, dyslipidemia, unhealthy diet, and smoking, can further amplify these pathways. Thus, MASLD and CAD should not be viewed as two isolated diseases with overlapping risk factors (34–41, 112). Rather, they may reinforce each other through a cascade involving insulin resistance, atherogenic dyslipidemia, lipotoxicity, oxidative stress, chronic inflammation, endothelial dysfunction, and genetic predisposition. These interacting pathways are summarized in Figure 1.

**Figure 2:**
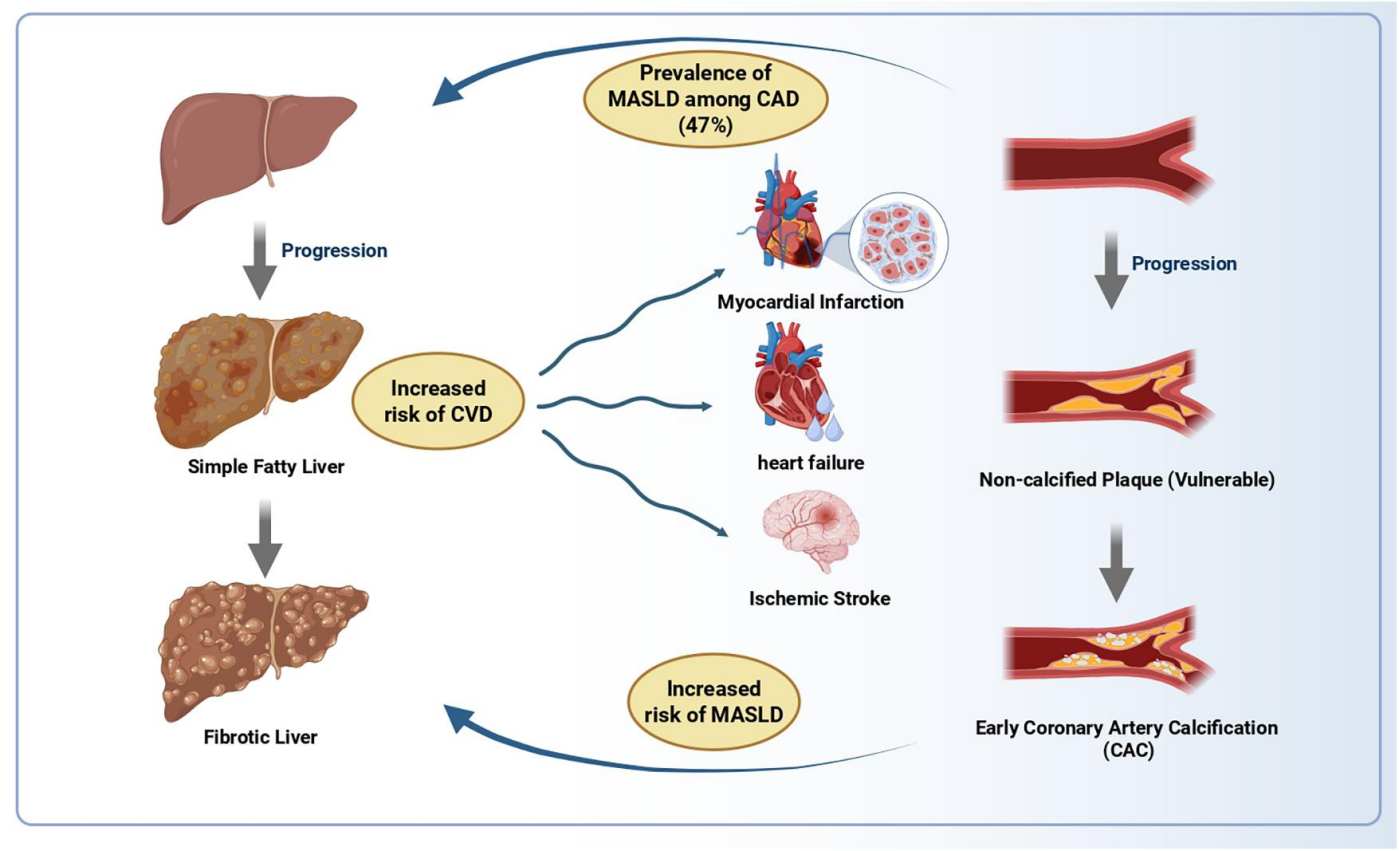
Epidemiological association and prognostic impact of MAFLD on CAD: A dual-pathway risk diagram. This schematic diagram summarizes the bidirectional epidemiological association and prognostic impact between metabolic dysfunction-associated steatotic liver disease (MASLD) and coronary artery disease (CAD). MASLD is prevalent in approximately 47% of CAD patients and heightens their risk of cardiovascular disease, including myocardial infarction, heart failure, ischemic stroke, and the formation of vulnerable non-calcified plaques. Conversely, CAD elevates the risk of developing MASLD and may promote both early coronary artery calcification and the progression of liver disease from simple steatosis to fibrosis.

## 4 Evidence for dual benefits of emerging pharmacotherapies

### 4.1 Resmetirom

Resmetirom is the first drug for MASH to achieve success in a phase III trial, as demonstrated in the MAESTRO-NASH study. This liver-specific thyroid hormone receptor-β agonist selectively activates hepatic thyroid hormone receptor β. Its mechanism effectively reduces hepatic steatosis and improves histopathological features. The treatment also mitigates markers of liver injury and dyslipidemia (13, 113).

The phase 3 MAESTRO-NASH trial found resmetirom superior to placebo in patients with biopsy-confirmed NASH. At doses of 80 mg and 100 mg, resmetirom significantly improved liver fibrosis (24.2–25.9% vs. 14.2% with placebo) and achieved NASH resolution (25.9–29.9% vs. 9.7%), with both outcomes reaching statistical significance (*P* < 0.001) (13).

Recent studies employing human liver-derived organoids demonstrate that Resmetirom directly and dose-dependently reduces lipid accumulation while elevating ATP levels (15). This effect likely involves mechanisms that promote lipolysis, enhance fatty acid *β*-oxidation, and improve mitochondrial function, which collectively reduce hepatic fat accumulation (14). This provides experimental evidence for its direct pharmacological mechanism in improving hepatic steatosis and mitochondrial function in MASLD/MASH treatment.

Clinical trials demonstrated that resmetirom had neutral effects on body weight and glucose metabolism, but it produced prominent improvements in lipid profiles, including LDL-C, triglycerides, and Lp(a) (13). Mendelian randomization indicated that these changes could reduce major cardiovascular events by 16%–21%, although long-term follow-up remains necessary to confirm clinical benefits and evaluate potential cardiovascular effects mediated by THR-α (113, 114).

### 4.2 GLP-1 receptor agonists and glucagon/GIP/GLP-1 receptor dual or triple agonists

A large body of research has confirmed that insulin resistance is associated with MASLD, obesity, and type 2 diabetes. There are already antidiabetic agents targeting insulin resistance, adipose tissue dysfunction, and the gut-insulinotropic system for the treatment of MASLD (115).

In recent years, novel multi-receptor agonists, including GLP-1RAs (116), have emerged as promising therapies for metabolic disorders, including MASLD, MASH, diabetes, and obesity. These drugs include dual agonists targeting GLP-1 and GIP receptors (such as tirzepatide) (117), dual agonists targeting GLP-1 and glucagon receptors (such as cotadutide) (17), and triple agonists that can act on all three receptors. These drugs have been proven to promote weight loss, improve blood glucose control, and improve lipid profiles (17). Recent retrospective studies indicate that the use of GLP-1RAs can improve adverse outcomes of MASLD, including liver decompensation, portal hypertension, hepatocellular carcinoma (HCC), or liver transplantation (LT) (118). In patients with MASLD who have not yet developed cirrhosis, GLP-1RA treatment significantly lowers the risk of fibrosis progression to cirrhosis, its related complications, and all-cause mortality (119).

Recent studies have also confirmed that GLP-1RAs significantly reduce the risk of major adverse cardiovascular events (MACE) by 14% or more (HR 0.86), lower all-cause mortality by 12%, and reduce the risk of heart failure-related hospitalizations by 11% (24, 116, 120). This may be related to weight loss, improved blood glucose control, and better lipid profiles (121, 122). These findings suggest that GLP-1 receptor agonists may reduce the risk of myocardial injury by preventing coronary artery occlusion, providing beneficial effects on microvascular diseases in the myocardium, or reducing myocardial damage caused by inflammation or other processes (116).

### 4.3 SGLT2 inhibitors

These drugs exert their hypoglycemic effect by inhibiting the sodium-glucose cotransporter 2 (SGLT2). Recent studies have shown that SGLT2 inhibitors offer not only glycemic control but also liver, kidney, and cardiovascular benefits beyond their effect on diabetes (123). In NAFLD patients, those who received SGLT2 inhibitors or GLP-1RAs showed similar effects on heart failure, major adverse cardiovascular events, cerebrovascular events, and all-cause mortality, all of which were superior to the control group (124, 125). SGLT2 inhibitors improve NAFLD by downregulating PFKFB3, inhibiting glycolysis, and modulating macrophage polarization (19). They can also improve non-alcoholic fatty liver disease-related fibrosis by downregulating miRNA-34a-5p, targeting GREM2 to inactivate hepatic stellate cells, lowering fasting blood glucose levels, liver-to-body weight ratio, ALT, AST levels, and inflammation (126). These inhibitors significantly improve insulin sensitivity, reduce liver fat content, and enhance blood glucose control and liver enzyme levels (125, 127).

Multiple studies have demonstrated that SGLT2 inhibitors directly exert anti-atherosclerotic and anti-inflammatory effects, manifested in the following ways: reducing infarct size, lowering systemic inflammation, inhibiting NLRP3 inflammasome activity (128), reducing local inflammation cell infiltration in the heart and adipose tissue, modulating macrophage polarization, and improving vascular function and cardiac remodeling (129). In post-myocardial infarction patients, regardless of whether they have diabetes or heart failure, SGLT2 inhibitors improve ventricular function recovery and reduce the risk of subsequent heart failure-related hospitalizations (123).

However, recent research has also shown that the differences in hsCRP, IL-6, neutrophil count, white blood cell count, NLR, and PLR levels between the empagliflozin and placebo groups at 26 weeks after myocardial infarction were not statistically significant (130), which challenges the hypothesis that its anti-inflammatory effects mediate cardiovascular protection.

The precise molecular mechanisms of SGLT2 inhibitors and their long-term safety and efficacy in broader populations, including non-diabetic patients, require further elucidation and validation through additional clinical data.

### 4.4 FGF21 analogues

FGF21, as a hepatic factor, significantly increases with the severity of MASLD and is correlated with indicators like liver fibrosis and inflammation (131). It has the potential to serve as a biomarker for assessing MASLD progression. However, FGF21 analogues have shown promise in improving the progression of MASLD. This may be due to FGF21 reducing hepatic lipid accumulation through both endocrine and autocrine signaling pathways, thereby inhibiting Kupffer cell activation, monocyte infiltration, and the accumulation of lipid or scar-related macrophages (132).

Many clinical drugs are currently under development.Clinical studies on the FGF21 analogue pegozafermin show that all dose groups experienced a significant reduction in liver fat fraction, while also improving liver function markers (ALT, AST) and lipid parameters (triglycerides, LDL-C, etc.), although changes in insulin resistance and HbA1c were not significant (98, 133). A study also found that in patients with moderate to severe fibrosis (F2-3) and cirrhosis (F4) due to MASH, the FGF21 analogue efruxifermin failed to significantly improve liver fibrosis in the short term, not reaching its primary endpoint (20). However, long-term (96 weeks) data indicated that high-dose (50 mg) treatment showed statistically significant fibrosis improvement, suggesting that the therapeutic effects might take longer to manifest (100). In contrast, another study demonstrated that after 24 weeks of Efruxifermin treatment, the treatment group had a significantly higher response rate compared to the placebo group (20%, *p* = 0.025 and *p* = 0.036) (100). It is certain that FGF21 analogues can improve clinical outcomes in MASLD patients.

A study examined the association between FGF21 levels and mortality in a population free of clinical cardiovascular disease. Their results indicated that before adjustment for confounding factors, FGF21 levels correlated with both cardiovascular and non-cardiovascular mortality. Following adjustment, however, this association remained significant only for non-cardiovascular mortality (134). FGF21 shows promise as a biomarker for long-term outcomes, although its association with cause-specific mortality requires further investigation. Another study found that in a population without clinical cardiovascular disease, higher FGF21 levels were significantly associated with the occurrence and progression of descending aortic calcification. However, the correlation with calcification in other key areas (such as coronary arteries and heart valves) was weak or not significant (135). Animal studies have confirmed that FGF21 significantly reduces plasma total cholesterol (especially non-HDL cholesterol) and accelerates the clearance of triglyceride-rich lipoproteins by activating brown adipose tissue (BAT) and inducing the browning of white adipose tissue (WAT), improving liver lipid metabolism, alleviating hepatic steatosis, reducing body fat, improving glucose tolerance, and positively affecting the area and stability of atherosclerotic plaques (136). Some specific mechanisms remain unclear, and further basic research and clinical trials are needed to confirm its cardiovascular effects.

### 4.5 PCSK9 inhibitors and statins

PCSK9 (Proprotein Convertase Subtilisin/Kexin Type 9) inhibitors and statins have been proven to significantly reduce MACE by lowering LDL-C levels. PCSK9 is a protein primarily secreted by the liver that regulates blood LDL cholesterol levels by binding to and promoting the degradation of LDL receptors (LDLR). High levels of PCSK9 lead to a reduction in LDLR, thereby increasing plasma LDL-C concentrations, which becomes a risk factor for coronary artery disease (CAD) (137). Recently developed PCSK9 inhibitors (such as monoclonal antibodies Alirocumab (138), Evolocumab (139), and lerodalcibep (140) block the interaction between PCSK9 and LDLR, significantly reducing LDL-C levels (138). These inhibitors offer new treatment options for patients with familial hypercholesterolemia or statin intolerance. Early use of Evolocumab can further reduce the risk of cardiovascular events, including cardiovascular death, myocardial infarction, stroke, and other composite endpoints, by 15%–23% (139). The novel oral PCSK9 inhibitor AZD0780, in combination with statin therapy, can effectively lower LDL-C by 35.3% to 50.7% within 12 weeks, with an adverse event rate similar to the placebo group (141).

Research by Holmes et al. indicates that PCSK9 gene variations were associated with lower LDL-C levels and significantly reduced the risk of carotid artery plaques, major occlusive vascular events, and ischemic stroke (142), further confirming the positive effects of PCSK9 inhibitors in treating cardiovascular diseases, including CAD.

However, the effect on MASLD appears to be negative. Animal studies have shown that while PCSK9 inhibitors lower plasma cholesterol by upregulating LDL receptors, they may also lead to excessive cholesterol accumulation in the liver, potentially increasing the risk of fatty liver, NASH, and liver cancer with long-term use (143). The study didn't evaluate how concurrent statin therapy influences these outcomes. Consequently, the clinical relevance of these findings remains to be substantiated through further investigation.

### 4.6 PPAR agonists

Peroxisome proliferator-activated receptor (PPAR) agonists are a potential therapeutic option for MASLD because they improve key metabolic abnormalities, including hepatic lipid accumulation, insulin resistance, and atherogenic dyslipidemia, which are also closely related to CAD risk (144, 145). By targeting these shared metabolic disturbances, PPAR agonists may be particularly useful for MASLD patients with type 2 diabetes, hypertriglyceridemia, or other cardiometabolic risk factors.

Among available agents, pioglitazone, a PPAR*γ* agonist, has the strongest clinical evidence in NASH/MASH, especially in patients with prediabetes or type 2 diabetes. Randomized trials have shown that pioglitazone can improve steatohepatitis activity and increase the likelihood of NASH resolution, although its effect on fibrosis is less consistent (146, 147). Its cardiovascular relevance is also notable. In high-risk patients with type 2 diabetes or insulin resistance, pioglitazone reduced some vascular outcomes, including myocardial infarction and stroke-related composite endpoint (148, 149). However, its use in CAD patients requires caution because weight gain, edema, and heart failure risk may limit its suitability, particularly in patients with established or suspected heart failure.

**Figure 3:**
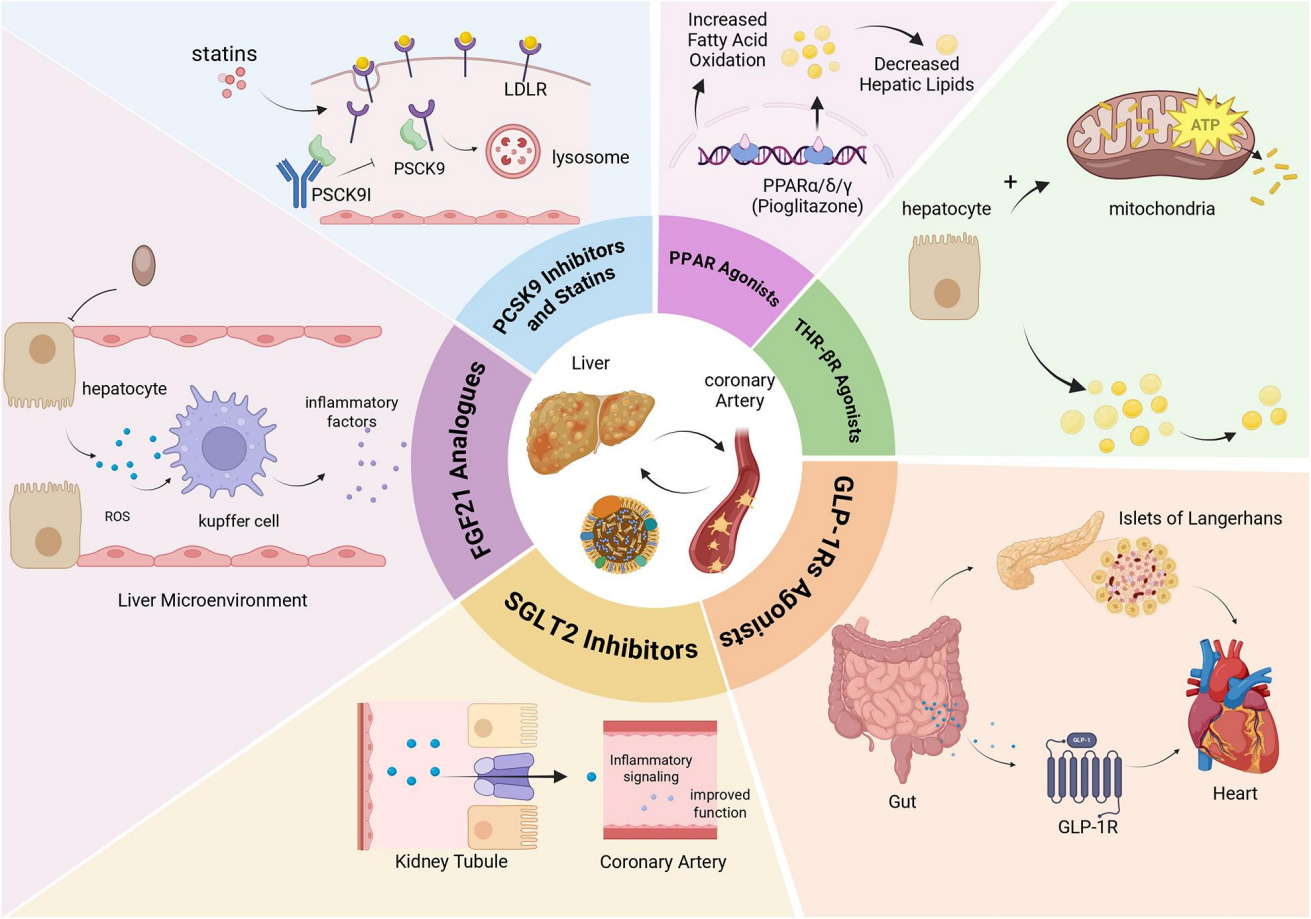
Multi-Organ mechanisms of action for current and emerging cardio-hepatic therapeutics. This schematic summarizes major pharmacological strategies that may reduce liver injury and cardiovascular risk in metabolic dysfunction-associated fatty liver disease (MAFLD). The central panel illustrates the liver–coronary arter*y* axis, highlighting interactions between hepatic metabolic dysfunction, circulating lipoproteins, and coronary vascular injury. PCSK9 inhibitors and statins improve lipid metabolism by regulating LDL receptor availability and reducing atherogenic lipoprotein burden. FGF21 analogues may attenuate hepatic oxidative stress, Kupffer cell activation, and inflammatory mediator release within the liver microenvironment. SGLT2 inhibitors act partly through renal metabolic effects and may improve coronary endothelial function and inflammatory signaling. GLP-1 receptor agonists exert systemic benefits through the gut–pancreatic islet–heart axis. THR-*β* agonists target hepatocytes to enhance mitochondrial activity, ATP production, and hepatic lipid clearance. Arrows indicate proposed mechanistic pathways or inter-organ communication. CAD: Coronary artery disease; FGF21: Fibroblast growth factor 21; GLP-1-R: Glucagon-like peptide-1 receptor; LDLR: Low-density lipoprotein receptor; PCSK9I/PCSK9 inhibitors: Proprotein convertase subtilisin/kexin type 9 inhibitors; ROS: Reactive oxygen species; SGLT2 inhibitors: Sodium-glucose cotransporter-2 inhibitors.

Newer dual or pan-PPAR agonists may offer broader metabolic effects. Lanifibranor, a pan-PPAR*α*/*δ*/*γ* agonist, improved histological disease activity in NASH and has also been associated with improvements in cardiometabolic markers in patients with MASH (150, 151). Saroglitazar, a dual PPAR*α*/*γ* agonist, improved liver enzymes, liver fat content, insulin resistance, and atherogenic dyslipidemia in patients with NAFLD/NASH (152). These findings suggest that PPAR agonists may help address both liver injury and cardiovascular risk factors in MASLD. However, direct evidence that MASLD-targeted PPAR agonists reduce CAD events remains limited. Experience with PPAR*α* drugs such as fenofibrate and pemafibrate also suggests that improving triglyceride-related lipid profiles does not always translate into fewer cardiovascular event (153). Thus, PPAR agonists should be viewed as a promising metabolic treatment strategy for selected MASLD patients, rather than as established CAD-protective therapy. Figure 3 summarizes major pharmacological strategies that may reduce liver injury and cardiovascular risk in metabolic dysfunction-associated fatty liver disease (MAFLD).

### 4.7 Hierarchy of clinical evidence for emerging heart–liver therapies

Although several drug classes have potential dual liver–cardiovascular relevance in MASLD, their levels of clinical evidence differ substantially. Resmetirom currently has the strongest liver-directed evidence, with phase 3 histological data showing improvement in MASH resolution and fibrosis-related endpoints in patients with NASH/MASH and moderate-to-advanced fibrosis (13). Semaglutide also has high-level evidence, as phase 3 data demonstrated significant histological benefit in MASH with F2–F3 fibrosis, and GLP-1 receptor agonist therapy has additional evidence for cardiovascular risk reduction in high-risk obesity or diabetes population (154, 155). However, not all GLP-1 receptor agonists have the same liver-related evidence, and the results should not be generalized across the whole drug class without caution.

SGLT2 inhibitors and PCSK9 inhibitors have stronger evidence for cardiovascular protection than for direct MASH treatment. SGLT2 inhibitors reduce cardiovascular and renal events in patients with type 2 diabetes or high cardiometabolic risk, and small randomized studies suggest that they may reduce liver fat in patients with type 2 diabetes and NAFLD (156–158). PCSK9 inhibitors have robust cardiovascular outcome evidence through LDL-C reduction, but their liver-related evidence remains limited mainly to mechanistic, observational, or early clinical data rather than biopsy-based MASH trial (159–161). Therefore, these agents should be considered cardiovascular risk-directed therapies with potential liver-related benefits, rather than established MASH-specific drugs.

By contrast, FGF21 analogues and PPAR agonists remain promising but less mature from a clinical implementation perspective. FGF21 analogues such as pegozafermin and efruxifermin have shown encouraging phase 2 histological or metabolic effects in NASH/MASH, but they lack completed cardiovascular outcome trials and are not yet established as routine clinical therapy (98, 100). PPAR agonists, including pioglitazone, lanifibranor, and saroglitazar, have shown benefits on steatohepatitis, liver fat, insulin sensitivity, or atherogenic dyslipidemia in selected trials, but their effects on fibrosis and cardiovascular outcomes are variable, and adverse effects such as weight gain, edema, or heart failure risk should be considered (146–148, 151, 152).

Overall, current evidence supports a phenotype-based approach: resmetirom or semaglutide may be prioritized for eligible MASH patients with significant fibrosis, GLP-1 receptor agonists or SGLT2 inhibitors for patients with obesity, type 2 diabetes, CKD, heart failure, or high cardiovascular risk, PCSK9 inhibitors for patients with severe atherogenic dyslipidemia or established CAD, and PPAR agonists for selected patients with insulin resistance or type 2 diabetes. Future trials should determine whether these therapies provide additive liver and cardiovascular protection when used alone or in rational combinations.

Table 2 shows the hierarchy of clinical evidence for drug classes with potential dual liver-cardiovascular effects in MASLD/MASH.

**Table 2:** Hierarchy of clinical evidence for drug classes with potential dual liver–cardiovascular effects in MASLD/MASH.

Drug	Level of liver-related evidence	Level of cardiovascular evidence	Conclusion
Resmetirom	High: phase 3 histological evidence; accelerated approval in the United States for non-cirrhotic MASH/NASH with F2–F3 fibrosis	Moderate: improves atherogenic lipid profiles, but lacks dedicated MACE outcome trials	A liver-directed MASH therapy with potential indirect cardiovascular benefits
GLP-1 receptor agonists	High: semaglutide has phase 3 histological evidence in MASH; evidence varies across different GLP-1 receptor agonists	High: several GLP-1 receptor agonists have demonstrated cardiovascular benefit in large outcome trials	Particularly relevant for MASLD/MASH patients with obesity, type 2 diabetes, or high cardiovascular risk
SGLT2 inhibitors	Low to moderate: small randomized trials and imaging-based studies suggest reductions in liver fat, but large biopsy-based MASH trials are lacking	High: cardiovascular and renal benefits are well established in patients with type 2 diabetes, heart failure, or CKD	Cardiometabolic risk-directed therapy rather than established MASH-specific treatment
FGF21 analogues	Moderate: phase 2/2b trials show promising histological or metabolic effects in NASH/MASH	Low to moderate: evidence is mainly based on surrogate markers such as lipid profile, body weight, and insulin sensitivity	Promising agents, but still under clinical development
PCSK9 inhibitors	Low: MASLD/MASH evidence is limited and mainly based on mechanistic, observational, or small clinical studies	High: FOURIER and ODYSSEY OUTCOMES support MACE reduction through LDL-C lowering	CAD and dyslipidemia-directed therapy; should not be described as established MASH treatment
PPAR agonists	Moderate: pioglitazone, lanifibranor, and saroglitazar have shown benefits in selected trials, but efficacy differs among agents	Moderate/inconsistent: pioglitazone has some vascular outcome signals, whereas PPAR*α*-related cardiovascular outcome data are mixed	May be useful in selected metabolic phenotypes, but evidence heterogeneity and safety concerns should be emphasized

### 4.8 Potential combination therapy for dual liver–cardiovascular protection

Because MASLD/MASH often appears with obesity, type 2 diabetes, dyslipidemia, CKD, heart failure, or established CAD, combination therapy may be useful for addressing both liver injury and cardiovascular risk. However, no fixed combination regimen has yet been proven in dedicated prospective trials to provide superior dual liver–cardiovascular protection. Thus, current combination strategies should be considered phenotype-driven approaches rather than established standard regimens.

A clinically relevant option is to combine liver-directed therapy with cardiometabolic agents. Resmetirom improves MASH-related histological endpoints and atherogenic lipid profiles, whereas GLP-1 receptor agonists improve body weight, glycemic control, insulin resistance, and cardiovascular risk (13, 155, 162, 163). Therefore, resmetirom plus a GLP-1 receptor agonist may be suitable for patients with MASH/MASLD complicated by obesity, type 2 diabetes, or high-risk/established CAD. A secondary analysis of MAESTRO-NASH suggested that background GLP-1 receptor agonist or SGLT2 inhibitor use did not reduce the efficacy of resmetirom, supporting the feasibility of such combinations, although this was not a dedicated combination-treatment trial (164).

Resmetirom plus an SGLT2 inhibitor may be more relevant for patients with type 2 diabetes, CKD, heart failure, or high cardiovascular risk, as SGLT2 inhibitors have established cardiovascular and renal benefits and may also reduce liver fat in patients with type 2 diabetes and NAFLD (156, 158, 165). In patients with established CAD, these therapies should be combined with standard cardiovascular prevention, including lipid-lowering therapy, antiplatelet therapy, blood pressure control, and glycemic management when clinically appropriate (26). Safety monitoring remains important, particularly for resmetirom-related hepatotoxicity, gallbladder events, and interactions with statins or CYP2C8/OATP inhibitors, as well as gastrointestinal effects of GLP-1 receptor agonists and genital infection, volume depletion, or rare ketoacidosis with SGLT2 inhibitors (155, 158, 162, 163, 165, 166). Overall, combination therapy is promising for selected MASLD/MASH patients with CAD or high cardiometabolic risk, but prospective studies are still needed to confirm its long-term liver and cardiovascular benefits.

## 5 Conclusion

Current evidence strongly indicates that cardiovascular risk assessment should accompany liver histological endpoints in patients with MASLD and MASH to enable comprehensive prognostic management. Consequently, future clinical practice should adopt a “dual-heart-liver” framework, where systematic cardiovascular risk screening and stratification become routine upon MASLD diagnosis, informing the development of individualized treatment strategies. This approach represents a fundamental shift from single-organ to multi-system integrated care.

For MASLD patients with high cardiovascular risk, GLP-1 receptor agonists—which randomized trials demonstrate improve liver histology while significantly reducing major adverse cardiovascular events—may be prioritized. Although resmetirom, as the first approved MASH-targeted drug, achieves notable liver pathology improvements, its long-term cardiovascular effects are not yet established. Future clinical drug studies should therefore incorporate both liver endpoints, such as histological remission and non-invasive tests, and cardiovascular endpoints, including major adverse cardiovascular events and plaque burden changes, to fully evaluate a therapy's value. Furthermore, growing clinical evidence supports combination regimens, pairing Resmetirom with established cardioprotective agents like GLP-1 receptor agonists or SGLT2 inhibitors, to maximize dual heart-liver protection and potentially mitigate monotherapy risks.

Artificial intelligence may further support future heart–liver research. In MASLD and CAD, machine learning and multi-omics approaches may help identify shared pathogenic targets and molecular subtypes (167, 168). And AI-based drug discovery platforms could facilitate virtual screening, lead compound optimization, and drug repurposing for agents with potential dual hepatic and cardiovascular benefits (169, 170). In clinical research, AI may also improve trial enrichment and patient selection by integrating liver disease severity, cardiometabolic features, imaging findings, and predicted treatment responses (171). However, these applications remain limited by data heterogeneity, insufficient external validation, and limited model interpretability. Therefore, AI should be regarded as a complementary tool for target discovery, drug development, and trial design, rather than a substitute for mechanistic studies and prospective clinical validation.

Despite the rationale for heart–liver co-management, several practical barriers still limit its routine implementation. First, MASLD and CAD are often managed separately by hepatologists, cardiologists, endocrinologists, and primary care physicians, which may lead to fragmented risk assessment and delayed intervention. Second, liver biopsy is not suitable for routine screening, whereas non-invasive tests require standardized use and careful interpretation across different clinical settings. Third, unified treatment algorithms for patients with both liver disease and cardiovascular disease remain limited, especially regarding drug prioritization, combination therapy, and safety monitoring. Finally, long-term follow-up systems that jointly track liver fibrosis, cardiometabolic risk factors, and cardiovascular outcomes are still underdeveloped. To address these gaps, future care should adopt multidisciplinary pathways, standardized stepwise risk stratification using non-invasive fibrosis and cardiovascular risk tools, phenotype-based drug selection, and integrated chronic disease follow-up systems. Such an approach may help translate the concept of dual heart–liver protection into routine clinical practice (7, 26, 112, 172).

Although pharmacological treatment has become an important focus in MASLD management, lifestyle modification remains the foundation of care. Weight reduction through dietary adjustment and regular physical activity can reduce hepatic steatosis, improve steatohepatitis, and may contribute to fibrosis regression when sufficient weight AHA/ACC/ACCP/ASPC/NLA/PCNA guideline for chronic coronary disease loss is achieved (25, 26, 173). Importantly, these interventions also address the cardiovascular dimension of MASLD by improving insulin resistance, body weight, blood pressure, lipid profiles, systemic inflammation, and cardiorespiratory fitness (7).

## References

[1] Rinella ME Lazarus JV Ratziu V Francque SM Sanyal AJ Kanwal F. A multisociety delphi consensus statement on new fatty liver disease nomenclature. Hepatology. (2023) 78(6):1966–86. 10.1097/HEP.0000000000000520 37363821 PMC10653297

[2] Ramírez-Mejía MM Jiménez-Gutiérrez C Eslam M George J Méndez-Sánchez N. Breaking new ground: mASLD vs. MAFLD-which holds the key for risk stratification? Hepatol Int. (2024) 18(1):168–78. 10.1007/s12072-023-10620-y 38127259

[3] Lazarus JV Mark HE Anstee QM Arab JP Batterham RL Castera L. Advancing the global public health agenda for NAFLD: a consensus statement. Nature reviews. Gastroenterol Hepatol (N Y). (2022) 19(1):60–78. 10.1038/s41575-021-00523-4 34707258

[4] Cho SMJ Koyama S Honigberg MC Surakka I Haidermota S Ganesh S. Genetic, sociodemographic, lifestyle, and clinical risk factors of recurrent coronary artery disease events: a population-based cohort study. Eur Heart J. (2023) 44(36):3456–65. 10.1093/eurheartj/ehad380 37350734 PMC10516626

[5] Younossi ZM Golabi P Paik JM Henry A Van Dongen C Henry L. The global epidemiology of nonalcoholic fatty liver disease (NAFLD) and nonalcoholic steatohepatitis (NASH): a systematic review. Hepatology. (2023) 77(4):1335–47. 10.1097/HEP.0000000000000004 36626630 PMC10026948

[6] Toh JZK Pan X-H Tay PWL Ng CH Yong JN Xiao J. A meta-analysis on the global prevalence, risk factors and screening of coronary heart disease in nonalcoholic fatty liver disease. Clin Gastroenterol Hepatol. (2022) 20(11):2462–2473.e10. 10.1016/j.cgh.2021.09.021 34560278

[7] Duell PB Welty FK Miller M Chait A Hammond G Ahmad Z. Nonalcoholic fatty liver disease and cardiovascular risk: a scientific statement from the American Heart Association. Arterioscler, Thromb, Vasc Biol. (2022) 42(6):e172–3. 10.1161/ATV.0000000000000153 35418240

[8] Ng CH Lim WH Hui Lim GE Hao Tan DJ Syn N Muthiah MD. Mortality outcomes by fibrosis stage in nonalcoholic fatty liver disease: a systematic review and meta-analysis. Clin Gastroenterol Hepatol. (2023) 21(4):931–939.e5. 10.1016/j.cgh.2022.04.014 35513235 PMC10792524

[9] Sanyal AJ Van Natta ML Clark J Neuschwander-Tetri BA Diehl AM Dasarathy S. Prospective study of outcomes in adults with nonalcoholic fatty liver disease. N Engl J Med. (2021) 385(17):1559–69. 10.1056/NEJMoa2029349 34670043 PMC8881985

[10] Montemezzo M AlTurki A Stahlschmidt F Olandoski M Rodrigo Tafarel J Precoma DB. Nonalcoholic fatty liver disease and coronary artery disease: big brothers in patients with acute coronary syndrome. TheScientificWorldJournal. (2020) 2020:8489238. 10.1155/2020/8489238 32327942 PMC7174950

[11] Munk Lauridsen M Ravnskjaer K Gluud LL Sanyal AJ. Disease classification, diagnostic challenges, and evolving clinical trial design in MASLD. J Clin Invest. (2025) 135(10):e189953. 10.1172/JCI189953 40371650 PMC12077896

[12] Ktenopoulos N Sagris M Gerogianni M Pamporis K Apostolos A Balampanis K. Non-alcoholic fatty liver disease and coronary artery disease: a bidirectional association based on endothelial dysfunction. Int J Mol Sci. (2024) 25(19):10595. 10.3390/ijms251910595 39408924 PMC11477211

[13] Harrison SA Bedossa P Guy CD Schattenberg JM Loomba R Taub R. A phase 3, randomized, controlled trial of resmetirom in NASH with liver fibrosis. N Engl J Med. (2024) 390(6):497–509. 10.1056/NEJMoa2309000 38324483

[14] Udompap P. Resmetirom: steatosis down, fibrosis out, peace of mind in. Hepatology. (2025) 81(4):1105–6. 10.1097/HEP.0000000000001245 40095856

[15] Li J Ayada I Pan Q. Resmetirom directly inhibits lipid accumulation in human liver-derived organoids. J Hepatol. (2025) 83(2):e86–7. 10.1016/j.jhep.2025.03.022 40147786

[16] Xiang L Wang G Zhuang Y Luo L Yan J Zhang H. Safety and efficacy of GLP-1/FGF21 dual agonist HEC88473 in MASLD and T2DM: a randomized, double-blind, placebo-controlled study. J Hepatol. (2025) 82(6):967–78. 10.1016/j.jhep.2024.12.006 39709140

[17] Harrison SA Browne SK Suschak JJ Tomah S Gutierrez JA Yang J. Effect of pemvidutide, a GLP-1/glucagon dual receptor agonist, on MASLD: a randomized, double-blind, placebo-controlled study. J Hepatol. (2025) 82(1):7–17. 10.1016/j.jhep.2024.07.006 39002641

[18] Rosén HC Mohammad MA Jernberg T James S Oldgren J Erlinge D. SGLT2 Inhibitors for patients with type 2 diabetes mellitus after myocardial infarction: a nationwide observation registry study from SWEDEHEART. Lancet Reg Health Eur. (2024) 45:101032. 10.1016/j.lanepe.2024.101032 39262451 PMC11387207

[19] Lin X-F Cui X-N Yang J Jiang Y-F Wei T-J Xia L. SGLT2 Inhibitors ameliorate NAFLD in mice via downregulating PFKFB3, suppressing glycolysis and modulating macrophage polarization. Acta Pharmacol Sin. (2024) 45(12):2579–97. 10.1038/s41401-024-01389-3 39294445 PMC11579449

[20] Noureddin M Rinella ME Chalasani NP Neff GW Lucas KJ Rodriguez ME. Efruxifermin in compensated liver cirrhosis caused by MASH. N Engl J Med. (2025) 392(24):2413–24. 10.1056/NEJMoa2502242 40341827

[21] Noureddin M Frias JP Neff GW Lucas KJ Behling C Bedossa P. Safety and efficacy of once-weekly efruxifermin versus placebo in metabolic dysfunction-associated steatohepatitis (HARMONY): 96-week results from a multicentre, randomised, double-blind, placebo-controlled, phase 2b trial. Lancet (London, England). (2025) 406(10504):719–30. 10.1016/S0140-6736(25)01073-6 40818852

[22] Zhang X Lau HCH Yu J. Pharmacological treatment for metabolic dysfunction-associated steatotic liver disease and related disorders: current and emerging therapeutic options. Pharmacol Rev. (2025) 77(2):100018. 10.1016/j.pharmr.2024.100018 40148030

[23] Bangalore S Maron DJ O’Brien SM Fleg JL Kretov EI Briguori C. Management of coronary disease in patients with advanced kidney disease. N Engl J Med. (2020) 382(17):1608–18. 10.1056/NEJMoa1915925 32227756 PMC7274537

[24] Ryan DH Lingvay I Deanfield J Kahn SE Barros E Burguera B. Long-term weight loss effects of semaglutide in obesity without diabetes in the SELECT trial. Nat Med. (2024) 30(7):2049–57. 10.1038/s41591-024-02996-7 38740993 PMC11271387

[25] European Association For The Study Of The Liver (Easl), European Association For The Study Of Diabetes (Easd), European Association For The Study Of Obesity (Easo). EASL-EASD-EASO clinical practice guidelines on the management of metabolic dysfunction-associated steatotic liver disease (MASLD). J Hepatol. (2024) 81(3):492–542. 10.1016/j.jhep.2024.04.031 38851997

[26] Virani SS Newby LK Arnold SV Bittner V Brewer LPC Demeter SH. 2023 AHA/ACC/ACCP/ASPC/NLA/PCNA guideline for the management of patients with chronic coronary disease: a report of the American Heart Association/American College of Cardiology joint committee on clinical practice guidelines. Circulation. (2023) 148(9):e9–e119. 10.1161/CIR.0000000000001168 37471501

[27] Vrints C Andreotti F Koskinas KC Rossello X Adamo M Ainslie J. 2024 ESC guidelines for the management of chronic coronary syndromes. Eur Heart J. (2024) 45(36):3415–537. 10.1093/eurheartj/ehae177 39210710

[28] Ren Z Simons PIHG Wesselius A Stehouwer CDA Brouwers MCGJ. Relationship between NAFLD and coronary artery disease: a Mendelian randomization study. Hepatology. (2023) 77(1):230–8. 10.1002/hep.32534 35441719 PMC9970021

[29] Simon TG Roelstraete B Alkhouri N Hagström H Sundström J Ludvigsson JF. Cardiovascular disease risk in paediatric and young adult non-alcoholic fatty liver disease. Gut. (2023) 72(3):573–80. 10.1136/gutjnl-2022-328105 36522149

[30] Alon L Corica B Raparelli V Cangemi R Basili S Proietti M. Risk of cardiovascular events in patients with non-alcoholic fatty liver disease: a systematic review and meta-analysis. Eur J Prev Cardiol. (2022) 29(6):938–46. 10.1093/eurjpc/zwab212 34939092

[31] Clayton-Chubb D Roberts SK Majeed A Woods RL Tonkin AM Nelson MR. Associations between MASLD, atrial fibrillation, cardiovascular events, mortality and aspirin use in older adults. Geroscience. (2025) 47(1):1303–18. 10.1007/s11357-024-01435-2 39607592 PMC11872849

[32] Mantovani A Csermely A Petracca G Beatrice G Corey KE Simon TG. Non-alcoholic fatty liver disease and risk of fatal and non-fatal cardiovascular events: an updated systematic review and meta-analysis. Lancet Gastroenterol Hepatol. (2021) 6(11):903–13. 10.1016/S2468-1253(21)00308-3 34555346

[33] Au Yeung SL Borges MC Wong THT Lawlor DA Schooling CM. Evaluating the role of non-alcoholic fatty liver disease in cardiovascular diseases and type 2 diabetes: a Mendelian randomization study in europeans and east asians. Int J Epidemiol. (2023) 52(3):921–31. 10.1093/ije/dyac212 36367831 PMC10244054

[34] Flessa C-M Nasiri-Ansari N Kyrou I Leca BM Lianou M Chatzigeorgiou A. Genetic and diet-induced animal models for non-alcoholic fatty liver disease (NAFLD) research. Int J Mol Sci. (2022) 23(24):15791. 10.3390/ijms232415791 36555433 PMC9780957

[35] Lindén D Romeo S. Therapeutic opportunities for the treatment of NASH with genetically validated targets. J Hepatol. (2023) 79(4):1056–64. 10.1016/j.jhep.2023.05.007 37207913

[36] Lee E-H Lee J-H Kim D-Y Lee Y-S Jo Y Dao T. Loss of SREBP-1c ameliorates iron-induced liver fibrosis by decreasing lipocalin-2. Exp Mol Med. (2024) 56(4):1001–12. 10.1038/s12276-024-01213-2 38622198 PMC11058876

[37] Nisar T Arshad K Abbas Z Khan MA Safdar S Shaikh RS. Prevalence of GCKR rs1260326 variant in subjects with obesity associated NAFLD and T2DM: a case-control study in south punjab, Pakistan. J Obes. (2023) 2023:6661858. 10.1155/2023/6661858 37829557 PMC10567336

[38] Gobeil É Bourgault J Mitchell PL Houessou U Gagnon E Girard A. Genetic inhibition of angiopoietin-like protein-3, lipids, and cardiometabolic risk. Eur Heart J. (2024) 45(9):707–21. 10.1093/eurheartj/ehad845 38243829 PMC10906986

[39] Brouwers MCGJ Simons N Stehouwer CDA Isaacs A. Non-alcoholic fatty liver disease and cardiovascular disease: assessing the evidence for causality. Diabetologia. (2020) 63(2):253–60. 10.1007/s00125-019-05024-3 31713012 PMC6946734

[40] Saki S Saki N Poustchi H Malekzadeh R. Assessment of genetic aspects of non-alcoholic fatty liver and premature cardiovascular events. Middle East J Dig Dis. (2020) 12(2):65–88. 10.34172/mejdd.2020.166 32626560 PMC7320986

[41] Gariani K Jornayvaz FR. Pathophysiology of NASH in endocrine diseases. Endocr Connect. (2021) 10(2):R52–65. 10.1530/EC-20-0490 33449917 PMC7983516

[42] Lee SB Park G-M Lee J-Y Lee BU Park JH Kim BG. Association between non-alcoholic fatty liver disease and subclinical coronary atherosclerosis: an observational cohort study. J Hepatol. (2018) 68(5):1018–24. 10.1016/j.jhep.2017.12.012 29274406

[43] Simon TG Roelstraete B Hagström H Sundström J Ludvigsson JF. Non-alcoholic fatty liver disease and incident major adverse cardiovascular events: results from a nationwide histology cohort. Gut. (2022) 71(9):1867–75. 10.1136/gutjnl-2021-325724 34489307

[44] Carter J Heseltine TD Meah MN Tzolos E Kwiecinski J Doris M. Hepatosteatosis and atherosclerotic plaque at coronary CT angiography. Radiol Cardiothor Imaging. (2022) 4(2):e210260. 10.1148/ryct.210260 PMC9059242 35506136

[45] Ichikawa K Lim J McClelland RL Susarla S Krishnan S Benzing T. Impact of nonalcoholic hepatic steatosis on the warranty period of a coronary artery calcium score of 0: results from the multi-ethnic study of atherosclerosis. Circ Cardiovasc Imaging. (2024) 17(9):e016465. 10.1161/CIRCIMAGING.123.016465 39288206 PMC11410342

[46] Park HE Kwak M-S Kim D Kim M-K Cha M- Choi S-Y. Nonalcoholic fatty liver disease is associated with coronary artery calcification development: a longitudinal study. J Clin Endocrinol Metab. (2016) 101(8):3134–43. 10.1210/jc.2016-1525 27253666

[47] Friedrich-Rust M Schoelzel F Maier S Seeger F Rey J Fichtlscherer S. Severity of coronary artery disease is associated with non-alcoholic fatty liver dis-ease: a single-blinded prospective mono-center study. PLoS One. (2017) 12(10):e0186720. 10.1371/journal.pone.0186720 29073252 PMC5658076

[48] Boddi M Tarquini R Chiostri M Marra F Valente S Giglioli C. Nonalcoholic fatty liver in nondiabetic patients with acute coronary syndromes. Eur J Clin Investig. (2013) 43(5):429–38. 10.1111/eci.12065 23480577

[49] Wong VW-S Wong GL-H Yeung JC-L Fung CY-K Chan JK-L Chang ZH-Y. Long-term clinical outcomes after fatty liver screening in patients undergoing coronary angiogram: a prospective cohort study. Hepatology. (2016) 63(3):754–63. 10.1002/hep.28253 26406278

[50] Ağaç MT Korkmaz L Çavuşoğlu G Karadeniz AG Ağaç S Bektas H. Association between nonalcoholic fatty liver disease and coronary artery disease complexity in patients with acute coronary syndrome: a pilot study. Angiology. (2013) 64(8):604–8. 10.1177/0003319713479155 23439214

[51] Baharvand-Ahmadi B Sharifi K Namdari M. Prevalence of non-alcoholic fatty liver disease in patients with coronary artery disease. ARYA Atheroscler. (2016) 12(4):201–5. 28149317 PMC5266138

[52] Wong VW-S Wong GL-H Yip GW-K Lo AO-S Limquiaco J Chu WC-W. Coronary artery disease and cardiovascular outcomes in patients with non-alcoholic fatty liver disease. Gut. (2011) 60(12):1721–7. 10.1136/gut.2011.242016 21602530

[53] Noda T Kamiya K Hamazaki N Nozaki K Ichikawa T Yamashita M. The prevalence of metabolic dysfunction-associated fatty liver disease and its association with physical function and prognosis in patients with acute coronary syndrome. J Clin Med. (2022) 11(7):1847. 10.3390/jcm11071847 35407455 PMC8999802

[54] Liu H-H Cao Y-X Jin J-L Guo Y-L Zhu C-G Wu N-Q. Metabolic-associated fatty liver disease and major adverse cardiac events in patients with chronic coronary syndrome: a matched case-control study. Hepatol Int. (2021) 15(6):1337–46. 10.1007/s12072-021-10252-0 34626331

[55] Bhardwaj S Sharma V. Association of nonalcoholic fatty liver disease in angiographically proven cases of coronary artery disease. Ind J Clin Cardiol. (2026) 7(1):9–16. 10.1177/26324636251383229

[56] Vilar-Gomez E Calzadilla-Bertot L Wai-Sun Wong V Castellanos M Aller-de la Fuente R Metwally M. Fibrosis severity as a determinant of cause-specific mortality in patients with advanced nonalcoholic fatty liver disease: a multi-national cohort study. Gastroenterology. (2018) 155(2):443–57. 10.1053/j.gastro.2018.04.034 29733831

[57] Hagström H Nasr P Ekstedt M Hammar U Stål P Hultcrantz R. Fibrosis stage but not NASH predicts mortality and time to development of severe liver disease in biopsy-proven NAFLD. J Hepatol. (2017) 67(6):1265–73. 10.1016/j.jhep.2017.07.027 28803953

[58] Taylor RS Taylor RJ Bayliss S Hagström H Nasr P Schattenberg JM. Association between fibrosis stage and outcomes of patients with nonalcoholic fatty liver disease: a systematic review and meta-analysis. Gastroenterology. (2020) 158(6):1611–1625.e12. 10.1053/j.gastro.2020.01.043 32027911

[59] Dulai PS Singh S Patel J Soni M Prokop LJ Younossi Z. Increased risk of mortality by fibrosis stage in nonalcoholic fatty liver disease: systematic review and meta-analysis. Hepatology. (2017) 65(5):1557–65. 10.1002/hep.29085 28130788 PMC5397356

[60] Angulo P Kleiner DE Dam-Larsen S Adams LA Bjornsson ES Charatcharoenwitthaya P. Liver fibrosis, but No other histologic features, is associated with long-term outcomes of patients with nonalcoholic fatty liver disease. Gastroenterology. (2015) 149(2):389–397.e10. 10.1053/j.gastro.2015.04.043 25935633 PMC4516664

[61] Targher G Byrne CD Lonardo A Zoppini G Barbui C. Non-alcoholic fatty liver disease and risk of incident cardiovascular disease: a meta-analysis. J Hepatol. (2016) 65(3):589–600. 10.1016/j.jhep.2016.05.013 27212244

[62] Moon JH Jeong S Jang H Koo BK Kim W. Metabolic dysfunction-associated steatotic liver disease increases the risk of incident cardiovascular disease: a nationwide cohort study. eClinicalMed. (2023) 65:102292. 10.1016/j.eclinm.2023.102292 PMC10632413 37954905

[63] Lee H-H Lee HA Kim E-J Kim HY Kim HC Ahn SH. Metabolic dysfunction-associated steatotic liver disease and risk of cardiovascular disease. Gut. (2024) 73(3):533–40. 10.1136/gutjnl-2023-331003 37907259

[64] Shi R Li X Sun K Liu F Kang B Wang Y. Association between severity of nonalcoholic fatty liver disease and major adverse cardiovascular events in patients assessed by coronary computed tomography angiography. BMC Cardiovasc Disord. (2024) 24(1):267. 10.1186/s12872-024-03880-5 38773388 PMC11107064

[65] Elsheikh E Younoszai Z Otgonsuren M Hunt S Raybuck B Younossi ZM. Markers of endothelial dysfunction in patients with non-alcoholic fatty liver disease and coronary artery disease. J Gastroenterol Hepatol. (2014) 29(7):1528–34. 10.1111/jgh.12549 25587619

[66] Xu S Ilyas I Little PJ Li H Kamato D Zheng X. Endothelial dysfunction in atherosclerotic cardiovascular diseases and beyond: from mechanism to pharmacotherapies. Pharmacol Rev. (2021) 73(3):924–67. 10.1124/pharmrev.120.000096 34088867

[67] Vanhoutte PM. Endothelial dysfunction: the first step toward coronary arteriosclerosis. Circ J. (2009) 73(4):595–601. 10.1253/circj.CJ-08-1169 19225203

[68] Villanova N Moscatiello S Ramilli S Bugianesi E Magalotti D Vanni E. Endothelial dysfunction and cardiovascular risk profile in nonalcoholic fatty liver disease. Hepatology. (2005) 42(2):473–80. 10.1002/hep.20781 15981216

[69] Persico M Masarone M Damato A Ambrosio M Federico A Rosato V. Non alcoholic fatty liver disease and eNOS dysfunction in humans. BMC Gastroenterol. (2017) 17(1):35. 10.1186/s12876-017-0592-y 28264657 PMC5340006

[70] Francque SM Van Der Graaff D Kwanten WJ. Non-alcoholic fatty liver disease and cardiovascular risk: pathophysiological mechanisms and implications. J Hepatol. (2016) 65(2):425–43. 10.1016/j.jhep.2016.04.005 27091791

[71] Kasumov T Edmison JM Dasarathy S Bennett C Lopez R Kalhan SC. Plasma levels of asymmetric dimethylarginine in patients with biopsy-proven nonalcoholic fatty liver disease. Metab, Clin Exp. (2011) 60(6):776–81. 10.1016/j.metabol.2010.07.027 20869086 PMC3012158

[72] Ionescu VA Gheorghe G Bacalbasa N Diaconu CC. Metabolic dysfunction-associated steatotic liver disease: pathogenetic links to cardiovascular risk. Biomolecules. (2025) 15(2):163. 10.3390/biom15020163 40001466 PMC11852489

[73] Feng S Chen JW Shu XY Aihemaiti M Quan JW Lu L. Endothelial microparticles: a mechanosensitive regulator of vascular homeostasis and injury under shear stress. Front Cell Dev Biol. (2022) 10:980112. 10.3389/fcell.2022.980112 36172284 PMC9510576

[74] Rashidi S Bagherpour G Abbasi-Malati Z Khosrowshahi ND Chegeni SA Roozbahani G. Endothelial progenitor cells for fabrication of engineered vascular units and angiogenesis induction. Cell Prolif. (2024) 57(9):e13716. 10.1111/cpr.13716 39051852 PMC11503262

[75] Peyter A-C Armengaud J-B Guillot E Yzydorczyk C. Endothelial progenitor cells dysfunctions and cardiometabolic disorders: from mechanisms to therapeutic approaches. Int J Mol Sci. (2021) 22(13):6667. 10.3390/ijms22136667 34206404 PMC8267891

[76] Cazac G-D Lăcătușu C-M Mihai C Grigorescu E-D Onofriescu A Mihai B-M. New insights into non-alcoholic fatty liver disease and coronary artery disease: the liver-heart axis. Life (Basel, Switzerland). (2022) 12(8):1189. 10.3390/life12081189 36013368 PMC9410285

[77] Sookoian S Castaño GO Burgueño AL Rosselli MS Gianotti TF Mallardi P. Circulating levels and hepatic expression of molecular mediators of atherosclerosis in nonalcoholic fatty liver disease. Atherosclerosis. (2010) 209(2):585–91. 10.1016/j.atherosclerosis.2009.10.011 19896127

[78] Bravo M Raurell I Barberá A Hide D Gil M Estrella F. Synergic effect of atorvastatin and ambrisentan on sinusoidal and hemodynamic alterations in a rat model of NASH. Dis Model Mech. (2021) 14(5):dmm048884. 10.1242/dmm.048884 34014280 PMC8188885

[79] Chen R Hou W Zhang Q Kang R Fan X-G Tang D. Emerging role of high-mobility group box 1 (HMGB1) in liver diseases. Mol Med. (2013) 19(1):357–66. 10.2119/molmed.2013.00099 24306421 PMC3883963

[80] Lee C-O Li H-L Tsoi M-F Cheung C-L Cheung BMY. Association between the liver fat score (LFS) and cardiovascular diseases in the national health and nutrition examination survey 1999–2016. Ann Med. (2021) 53(1):1065–73. 10.1080/07853890.2021.1943514 34184611 PMC8245099

[81] Boutari C Mantzoros CS. Adiponectin and leptin in the diagnosis and therapy of NAFLD. Metabolism. (2020) 103:154028. 10.1016/j.metabol.2019.154028 31785257

[82] Okekunle AP Youn J Song S Chung GE Yang SY Kim YS. Predicted pro-inflammatory hs-CRP score and non-alcoholic fatty liver disease. Gastroenterol Rep (Oxf). (2023) 11:goad059. 10.1093/gastro/goad059 37842198 PMC10568523

[83] Lee KC Wu PS Lin HC. Pathogenesis and treatment of non-alcoholic steatohepatitis and its fibrosis. Clin Mol Hepatol. (2023) 29(1):77–98. 10.3350/cmh.2022.0237 36226471 PMC9845678

[84] Smith GI Shankaran M Yoshino M Schweitzer GG Chondronikola M Beals JW. Insulin resistance drives hepatic *de novo* lipogenesis in nonalcoholic fatty liver disease. J Clin Invest. (2020) 130(3):1453–60. 10.1172/JCI134165 31805015 PMC7269561

[85] Caussy C Aubin A Loomba R. The relationship between type 2 diabetes, NAFLD, and cardiovascular risk. Curr Diab Rep. (2021) 21(5):15. 10.1007/s11892-021-01383-7 33742318 PMC8805985

[86] Merino J Leong A Posner DC Porneala B Masana L Dupuis J. Genetically driven hyperglycemia increases risk of coronary artery disease separately from type 2 diabetes. Diabetes Care. (2017) 40(5):687–93. 10.2337/dc16-2625 28298470 PMC5399655

[87] Guo J Du L. An update on ox-LDL-inducing vascular smooth muscle cell-derived foam cells in atherosclerosis. Front Cell Dev Biol. (2024) 12:1481505. 10.3389/fcell.2024.1481505 39524227 PMC11543427

[88] Jiang H Zhou Y Nabavi SM Sahebkar A Little PJ Xu S. Mechanisms of oxidized LDL-mediated endothelial dysfunction and its consequences for the development of atherosclerosis. Front Cardiovasc Med. (2022) 9:925923. 10.3389/fcvm.2022.925923 35722128 PMC9199460

[89] Jebari-Benslaiman S Galicia-García U Larrea-Sebal A Olaetxea JR Alloza I Vandenbroeck K. Pathophysiology of atherosclerosis. Int J Mol Sci. (2022) 23(6):3346. 10.3390/ijms23063346 35328769 PMC8954705

[90] He B Copps KD Stöhr O Liu B Hu S Joshi S. Spatial regulation of glucose and lipid metabolism by hepatic insulin signaling. Cell Metab. (2025) 37(7):1568–1583.e7. 10.1016/j.cmet.2025.03.015 40245868 PMC12221773

[91] Anwar SD Foster C Ashraf A. Lipid disorders and metabolic-associated fatty liver disease. Endocrinol Metab Clin N Am. (2023) 52(3):445–57. 10.1016/j.ecl.2023.01.003 37495336

[92] Heeren J Scheja L. Metabolic-associated fatty liver disease and lipoprotein metabolism. Mol Metab. (2021) 50:101238. 10.1016/j.molmet.2021.101238 33892169 PMC8324684

[93] Nguyen M Asgharpour A Dixon DL Sanyal AJ Mehta A. Emerging therapies for MASLD and their impact on plasma lipids. Am J Prev Cardiol. (2024) 17:100638. 10.1016/j.ajpc.2024.100638 38375066 PMC10875196

[94] Zhao Y Liu L Yang S Liu G Pan L Gu C. Mechanisms of atherosclerosis induced by postprandial lipemia. Front Cardiovasc Med. (2021) 8:636947. 10.3389/fcvm.2021.636947 33996937 PMC8116525

[95] Yanai H Adachi H Hakoshima M Katsuyama H. Postprandial hyperlipidemia: its pathophysiology, diagnosis, atherogenesis, and treatments. Int J Mol Sci. (2023) 24(18):13942. 10.3390/ijms241813942 37762244 PMC10530470

[96] Deprince A Haas JT Staels B. Dysregulated lipid metabolism links NAFLD to cardiovascular disease. Mol Metab. (2020) 42:101092. 10.1016/j.molmet.2020.101092 33010471 PMC7600388

[97] Chapman M J Guérin M Bruckert E. Atherogenic, dense low-density lipoproteins. Pathophysiology and new therapeutic approaches. Eur Heart J. (1998) 19(Suppl A):A24–30. 9519339

[98] Loomba R Sanyal AJ Kowdley KV Bhatt DL Alkhouri N Frias JP. Randomized, controlled trial of the FGF21 analogue pegozafermin in NASH. N Engl J Med. (2023) 389(11):998–1008. 10.1056/NEJMoa2304286 37356033 PMC10718287

[99] Wu Y-W Chang T-T Chang C-C Chen J-W. Fatty-acid-binding protein 4 as a novel contributor to mononuclear cell activation and endothelial cell dysfunction in atherosclerosis. Int J Mol Sci. (2020) 21(23):9245. 10.3390/ijms21239245 33287461 PMC7730098

[100] Harrison SA Frias JP Neff G Abrams GA Lucas KJ Sanchez W. Safety and efficacy of once-weekly efruxifermin versus placebo in non-alcoholic steatohepatitis (HARMONY): a multicentre, randomised, double-blind, placebo-controlled, phase 2b trial. Lancet Gastroenterol Hepatol. (2023) 8(12):1080–93. 10.1016/S2468-1253(23)00272-8 37802088

[101] Yoon MY Sung JM Song CS Lee WY Rhee EJ Shin JH. Enhanced a-FABP expression in visceral fat: potential contributor to the progression of NASH. Clin Mol Hepatol. (2012) 18(3):279–86. 10.3350/cmh.2012.18.3.279 23091808 PMC3467431

[102] Chen Z Tian R She Z Cai J Li H. Role of oxidative stress in the pathogenesis of nonalcoholic fatty liver disease. Free Radical Biol Med. (2020) 152:116–41. 10.1016/j.freeradbiomed.2020.02.025 32156524

[103] Arroyave-Ospina JC Wu Z Geng Y Moshage H. Role of oxidative stress in the pathogenesis of non-alcoholic fatty liver disease: implications for prevention and therapy. Antioxidants. (2021) 10(2):174. 10.3390/antiox10020174 33530432 PMC7911109

[104] Karkucinska-Wieckowska A Simoes ICM Kalinowski P Lebiedzinska-Arciszewska M Zieniewicz K Milkiewicz P. Mitochondria, oxidative stress and nonalcoholic fatty liver disease: a complex relationship. Eur J Clin Investig. (2022) 52(3):e13622. 10.1111/eci.13622 34050922

[105] Rives C Fougerat A Ellero-Simatos S Loiseau N Guillou H Gamet-Payrastre L. Oxidative stress in NAFLD: role of nutrients and food contaminants. Biomolecules. (2020) 10(12):1702. 10.3390/biom10121702 33371482 PMC7767499

[106] Hoebinger C Rajcic D Hendrikx T. Oxidized lipids: common immunogenic drivers of non-alcoholic fatty liver disease and atherosclerosis. Front Cardiovasc Med. (2022) 8:824481. 10.3389/fcvm.2021.824481 35083304 PMC8784685

[107] Batty M Bennett MR Yu E. The role of oxidative stress in atherosclerosis. Cells. (2022) 11(23):3843. 10.3390/cells11233843 36497101 PMC9735601

[108] Mury P Chirico EN Mura M Millon A Canet-Soulas E Pialoux V. Oxidative stress and inflammation, key targets of atherosclerotic plaque progression and vulnerability: potential impact of physical activity. Sports Medicine). (2018) 48(12):2725–41. 10.1007/s40279-018-0996-z 30302720

[109] Tacke F Puengel T Loomba R Friedman SL. An integrated view of anti-inflammatory and antifibrotic targets for the treatment of NASH. J Hepatol. (2023) 79(2):552–66. 10.1016/j.jhep.2023.03.038 37061196 PMC12164378

[110] Hammerich L Tacke F. Hepatic inflammatory responses in liver fibrosis. Nat Rev Gastroenterol Hepatol. (2023) 20(10):633–46. 10.1038/s41575-023-00807-x 37400694

[111] Hsu P-F Wang Y-W Lin C-C Wang Y-J Ding Y-Z Liou T-L. The association of the steatosis severity in fatty liver disease with coronary plaque pattern in general population. Liver Int. (2021) 41(1):81–90. 10.1111/liv.14637 33373113

[112] Visseren FLJ Mach F Smulders YM Carballo D Koskinas KC Bäck M. 2021 ESC guidelines on cardiovascular disease prevention in clinical practice. Eur Heart J. (2021) 42(34):3227–337. 10.1093/eurheartj/ehab484 34458905

[113] Harrison SA Taub R Neff GW Lucas KJ Labriola D Moussa SE. Resmetirom for nonalcoholic fatty liver disease: a randomized, double-blind, placebo-controlled phase 3 trial. Nat Med. (2023) 29(11):2919–28. 10.1038/s41591-023-02603-1 37845512 PMC10667098

[114] Petta S Targher G Romeo S Pajvani UB Zheng M-H Aghemo A. The first MASH drug therapy on the horizon: current perspectives of resmetirom. Liver Int. (2024) 44(7):1526–36. 10.1111/liv.15930 38578141

[115] Ciccarelli G Di Giuseppe G Cinti F Moffa S Mezza T Giaccari A. Why do some glucose-lowering agents improve non-alcoholic fatty liver disease whereas others do not? A narrative review in search of a unifying hypothesis. Diabetes Metab Res Rev. (2023) 39(7):e3668. 10.1002/dmrr.3668 37309298

[116] Zafer M Tavaglione F Romero-Gómez M Loomba R. Review article: gLP-1 receptor agonists and glucagon/GIP/GLP-1 receptor dual or triple agonists—mechanism of action and emerging therapeutic landscape in MASLD. Aliment Pharmacol Ther. (2025) 61(12):1872–88. 10.1111/apt.70196 40364529 PMC12323726

[117] Wilson JM Nikooienejad A Robins DA Roell WC Riesmeyer JS Haupt A. The dual glucose-dependent insulinotropic peptide and glucagon-like peptide-1 receptor agonist, tirzepatide, improves lipoprotein biomarkers associated with insulin resistance and cardiovascular risk in patients with type 2 diabetes. Diabetes, Obes Metab. (2020) 22(12):2451–59. 10.1111/dom.14174 33462955 PMC7756479

[118] Elsaid MI Li N Firkins SA Rustgi VK Paskett ED Acharya C. Impacts of glucagon-like peptide-1 receptor agonists on the risk of adverse liver outcomes in patients with metabolic dysfunction-associated steatotic liver disease cirrhosis and type 2 diabetes. Aliment Pharmacol Ther. (2024) 59(9):1096–110. 10.1111/apt.17925 38538967

[119] Kanwal F Kramer JR Li L Yang Y-X Cao Y Yu X. GLP-1 receptor agonists and risk for cirrhosis and related complications in patients with metabolic dysfunction-associated steatotic liver disease. JAMA Intern Med. (2024) 184(11):1314–23. 10.1001/jamainternmed.2024.4661 39283612 PMC11406452

[120] Sattar N Lee MMY Kristensen SL Branch KRH Del Prato S Khurmi NS. Cardiovascular, mortality, and kidney outcomes with GLP-1 receptor agonists in patients with type 2 diabetes: a systematic review and meta-analysis of randomised trials. Lancet Diabetes Endocrinol. (2021) 9(10):653–62. 10.1016/S2213-8587(21)00203-5 34425083

[121] Targher G Mantovani A Byrne CD. Mechanisms and possible hepatoprotective effects of glucagon-like peptide-1 receptor agonists and other incretin receptor agonists in non-alcoholic fatty liver disease. Lancet Gastroenterol Hepatol. (2023) 8(2):179–91. 10.1016/S2468-1253(22)00338-7 36620987

[122] Fisman EZ Tenenbaum A. The dual glucose-dependent insulinotropic polypeptide (GIP) and glucagon-like peptide-1 (GLP-1) receptor agonist tirzepatide: a novel cardiometabolic therapeutic prospect. Cardiovasc Diabetol. (2021) 20(1):225. 10.1186/s12933-021-01412-5 34819089 PMC8613929

[123] Cuttone A Cannavò V Abdullah RMS Fugazzotto P Arena G Brancati S. Expanding the use of SGLT2 inhibitors in T2D patients across clinical settings. Cells. (2025) 14(9):668. 10.3390/cells14090668 40358192 PMC12071329

[124] Krishnan A Schneider CV Hadi Y Mukherjee D AlShehri B Alqahtani SA. Cardiovascular and mortality outcomes with GLP-1 receptor agonists vs other glucose-lowering drugs in individuals with NAFLD and type 2 diabetes: a large population-based matched cohort study. Diabetologia. (2024) 67(3):483–93. 10.1007/s00125-023-06057-5 38117293 PMC10844347

[125] Mantovani A Byrne CD Targher G. Efficacy of peroxisome proliferator-activated receptor agonists, glucagon-like peptide-1 receptor agonists, or sodium-glucose cotransporter-2 inhibitors for treatment of non-alcoholic fatty liver disease: a systematic review. Lancet Gastroenterol Hepatol. (2022) 7(4):367–78. 10.1016/S2468-1253(21)00261-2 35030323

[126] Shen Y Cheng L Xu M Wang W Wan Z Xiong H. SGLT2 Inhibitor empagliflozin downregulates miRNA-34a-5p and targets GREM2 to inactivate hepatic stellate cells and ameliorate non-alcoholic fatty liver disease-associated fibrosis. Metab, Clin Exp. (2023) 146:155657. 10.1016/j.metabol.2023.155657 37422021

[127] Borisov AN Kutz A Christ ER Heim MH Ebrahimi F. Canagliflozin and metabolic associated fatty liver disease in patients with diabetes mellitus: new insights from CANVAS. J Clin Endocrinol Metab. (2023) 108(11):2940–9. 10.1210/clinem/dgad249 37149821 PMC10584001

[128] Moon JS Hong JH Jung YJ Ferrannini E Nauck MA Lim S. SGLT-2 inhibitors and GLP-1 receptor agonists in metabolic dysfunction-associated fatty liver disease. Trends Endocrinol Metab. (2022) 33(6):424–42. 10.1016/j.tem.2022.03.005 35491295

[129] Paolisso P Bergamaschi L Santulli G Gallinoro E Cesaro A Gragnano F. Infarct size, inflammatory burden, and admission hyperglycemia in diabetic patients with acute myocardial infarction treated with SGLT2-inhibitors: a multicenter international registry. Cardiovasc Diabetol. (2022) 21(1):77. 10.1186/s12933-022-01506-8 35570280 PMC9107763

[130] Benedikt M Mangge H Aziz F Curcic P Pailer S Herrmann M. Impact of the SGLT2-inhibitor empagliflozin on inflammatory biomarkers after acute myocardial infarction—a *post-hoc* analysis of the EMMY trial. Cardiovasc Diabetol. (2023) 22(1):166. 10.1186/s12933-023-01904-6 37407956 PMC10324245

[131] Gallego-Durán R Ampuero J Maya-Miles D Pastor-Ramírez H Montero-Vallejo R Rivera-Esteban J. Fibroblast growth factor 21 is a hepatokine involved in MASLD progression. United European Gastroenterol J. (2024) 12(8):1056–68. 10.1002/ueg2.12534 PMC11485537 38894596

[132] Liu C Schönke M Spoorenberg B Lambooij JM Van Der Zande HJP Zhou E. FGF21 Protects against hepatic lipotoxicity and macrophage activation to attenuate fibrogenesis in nonalcoholic steatohepatitis. Elife. (2023) 12:e83075. 10.7554/eLife.83075 36648330 PMC9928421

[133] Loomba R Lawitz EJ Frias JP Ortiz-Lasanta G Johansson L Franey BB. Safety, pharmacokinetics, and pharmacodynamics of pegozafermin in patients with non-alcoholic steatohepatitis: a randomised, double-blind, placebo-controlled, phase 1b/2a multiple-ascending-dose study. Lancet Gastroenterol Hepatol. (2023) 8(2):120–32. 10.1016/S2468-1253(22)00347-8 36521501

[134] Tucker WJ Tucker B Januszewski AS Jenkins AJ Keech AC Kestenbaum BR. Association of circulating fibroblast growth factor 21 levels with all-cause and cardiovascular mortality: the multi-ethnic study of atherosclerosis. Clin Chim Acta. (2024) 555:117799. 10.1016/j.cca.2024.117799 38309558

[135] Sayanthan S Allison MA Budoff MJ Rye K-A Ong KL. Relationship of fibroblast growth factor 21 with the prevalence and progression of vascular and valvular calcification: multi-ethnic study of atherosclerosis. Int J Cardiol. (2023) 370:388–95. 10.1016/j.ijcard.2022.10.145 36306948

[136] Liu C Schönke M Zhou E Li Z Kooijman S Boon MR. Pharmacological treatment with FGF21 strongly improves plasma cholesterol metabolism to reduce atherosclerosis. Cardiovasc Res. (2022) 118(2):489–502. 10.1093/cvr/cvab076 33693480 PMC8803070

[137] Kestecher BM Németh K Ghosal S Sayour NV Gergely TG Bodnár BR. Reduced circulating CD63 + extracellular vesicle levels associate with atherosclerosis in hypercholesterolaemic mice and humans. Cardiovasc Diabetol. (2024) 23(1):368. 10.1186/s12933-024-02459-w 39420340 PMC11487797

[138] Räber L Ueki Y Otsuka T Losdat S Häner JD Lonborg J. Effect of alirocumab added to high-intensity statin therapy on coronary atherosclerosis in patients with acute myocardial infarction: the PACMAN-AMI randomized clinical trial. JAMA. (2022) 327(18):1771–81. 10.1001/jama.2022.5218 35368058 PMC8978048

[139] O’Donoghue ML Giugliano RP Wiviott SD Atar D Keech A Kuder JF. Long-term evolocumab in patients with established atherosclerotic cardiovascular disease. Circulation. (2022) 146(15):1109–19. 10.1161/CIRCULATIONAHA.122.061620 36031810

[140] O’Donoghue ML Fazio S Giugliano RP Stroes ESG Kanevsky E Gouni-Berthold I. Lipoprotein(a), PCSK9 inhibition, and cardiovascular risk. Circulation. (2019) 139(12):1483–92. 10.1161/CIRCULATIONAHA.118.037184 30586750

[141] Koren MJ Vega RB Agrawal N Xu Y Barbour AM Yu H. An oral PCSK9 inhibitor for treatment of hypercholesterolemia: the PURSUIT randomized trial. J Am Coll Cardiol. (2025) 85(21):1996–2007. 10.1016/j.jacc.2025.03.499 40167413

[142] Holmes MV Kartsonaki C Boxall R Lin K Reeve N Yu C. PCSK9 Genetic variants and risk of vascular and non-vascular diseases in Chinese and UK populations. Eur J Prev Cardiol. (2024) 31(8):1015–25. 10.1093/eurjpc/zwae009 38198221 PMC11144468

[143] Ioannou GN Lee SP Linsley PS Gersuk V Yeh MM Chen Y-Y. Pcsk9 deletion promotes murine nonalcoholic steatohepatitis and hepatic carcinogenesis: role of cholesterol. Hepatol Commun. (2022) 6(4):780–94. 10.1002/hep4.1858 34816633 PMC8948564

[144] Francque S Szabo G Abdelmalek MF Byrne CD Cusi K Dufour J-F. Nonalcoholic steatohepatitis: the role of peroxisome proliferator-activated receptors. Nat Rev Gastroenterol Hepatol. (2021) 18(1):24–39. 10.1038/s41575-020-00366-5 33093663

[145] Staels B Butruille L Francque S. Treating NASH by targeting peroxisome proliferator-activated receptors. J Hepatol. (2023) 79(5):1302–16. 10.1016/j.jhep.2023.07.004 37459921

[146] Sanyal AJ Chalasani N Kowdley KV McCullough A Diehl AM Bass NM. Pioglitazone, vitamin E, or placebo for nonalcoholic steatohepatitis. N Engl J Med. (2010) 362(18):1675–85. 10.1056/NEJMoa0907929 20427778 PMC2928471

[147] Cusi K Orsak B Bril F Lomonaco R Hecht J Ortiz-Lopez C. Long-term pioglitazone treatment for patients with nonalcoholic steatohepatitis and prediabetes or type 2 diabetes mellitus: a randomized trial. Ann Intern Med. (2016) 165(5):305–15. 10.7326/M15-1774 27322798

[148] Dormandy JA Charbonnel B Eckland DJA Erdmann E Massi-Benedetti M Moules IK. Secondary prevention of macrovascular events in patients with type 2 diabetes in the PROactive study (PROspective pioglitAzone clinical trial in macroVascular events): a randomised controlled trial. Lancet. (2005) 366(9493):1279–89. 10.1016/S0140-6736(05)67528-9 16214598

[149] Kernan WN Viscoli CM Furie KL Young LH Inzucchi SE Gorman M. Pioglitazone after ischemic stroke or transient ischemic attack. N Engl J Med. (2016) 374(14):1321–31. 10.1056/NEJMoa1506930 26886418 PMC4887756

[150] Cooreman MP Butler J Giugliano RP Zannad F Dzen L Huot-Marchand P. The pan-PPAR agonist lanifibranor improves cardiometabolic health in patients with metabolic dysfunction-associated steatohepatitis. Nat Commun. (2024) 15(1):3962. 10.1038/s41467-024-47919-9 38730247 PMC11087475

[151] Francque SM Bedossa P Ratziu V Anstee QM Bugianesi E Sanyal AJ. A randomized, controlled trial of the pan-PPAR agonist lanifibranor in NASH. N Engl J Med. (2021) 385(17):1547–58. 10.1056/NEJMoa2036205 34670042

[152] Gawrieh S Noureddin M Loo N Mohseni R Awasty V Cusi K. Saroglitazar, a PPAR-α/*γ* agonist, for treatment of NAFLD: a randomized controlled double-blind phase 2 trial. Hepatology. (2021) 74(4):1809–24. 10.1002/hep.31843 33811367

[153] Das Pradhan A Glynn RJ Fruchart JC MacFadyen JG Zaharris ES Everett BM. Triglyceride lowering with pemafibrate to reduce cardiovascular risk. N Engl J Med. (2022) 387(21):1923–34. 10.1056/NEJMoa2210645 36342113

[154] Sanyal AJ Newsome PN Kliers I Østergaard LH Long MT Kjær MS. Phase 3 trial of semaglutide in metabolic dysfunction-associated steatohepatitis. N Engl J Med. (2025) 392(21):2089–99. 10.1056/NEJMoa2413258 40305708

[155] Lincoff AM Brown-Frandsen K Colhoun HM Deanfield J Emerson SS Esbjerg S. Semaglutide and cardiovascular outcomes in obesity without diabetes. N Engl J Med. (2023) 389(24):2221–32. 10.1056/NEJMoa2307563 37952131

[156] Zinman B Wanner C Lachin JM Fitchett D Bluhmki E Hantel S. Empagliflozin, cardiovascular outcomes, and mortality in type 2 diabetes. N Engl J Med. (2015) 373(22):2117–28. 10.1056/NEJMoa1504720 26378978

[157] Wiviott SD Raz I Bonaca MP Mosenzon O Kato ET Cahn A. Dapagliflozin and cardiovascular outcomes in type 2 diabetes. N Engl J Med. (2019) 380(4):347–57. 10.1056/NEJMoa1812389 30415602

[158] Kuchay MS Krishan S Mishra SK Farooqui KJ Singh MK Wasir JS. Effect of empagliflozin on liver fat in patients with type 2 diabetes and nonalcoholic fatty liver disease: a randomized controlled trial (E-LIFT trial). Diabetes Care. (2018) 41(8):1801–8. 10.2337/dc18-0165 29895557

[159] Sabatine MS Giugliano RP Keech AC Honarpour N Wiviott SD Murphy SA. Evolocumab and clinical outcomes in patients with cardiovascular disease. N Engl J Med. (2017) 376(18):1713–22. 10.1056/NEJMoa1615664 28304224

[160] Schwartz GG Steg PG Szarek M Bhatt DL Bittner VA Diaz R. Alirocumab and cardiovascular outcomes after acute coronary syndrome. N Engl J Med. (2018) 379(22):2097–107. 10.1056/NEJMoa1801174 30403574

[161] Momtazi-Borojeni AA Banach M Ruscica M Sahebkar A. The role of PCSK9 in NAFLD/NASH and therapeutic implications of PCSK9 inhibition. Expert Rev Clin Pharmacol. (2022) 15(10):1199–208. 10.1080/17512433.2022.2132229 36193738

[162] Newsome PN Buchholtz K Cusi K Linder M Okanoue T Ratziu V. A placebo-controlled trial of subcutaneous semaglutide in nonalcoholic steatohepatitis. N Engl J Med. (2021) 384(12):1113–24. 10.1056/NEJMoa2028395 33185364

[163] Loomba R Hartman ML Lawitz EJ Vuppalanchi R Boursier J Bugianesi E. Tirzepatide for metabolic dysfunction-associated steatohepatitis with liver fibrosis. N Engl J Med. (2024) 391(4):299–310. 10.1056/NEJMoa2401943 38856224

[164] Noureddin M Rinella M Taub R Labriola D Camacho RC Alkhouri N. Effects of resmetirom on metabolic-dysfunction associated steatohepatitis in patients with weight loss and/or diabetes taking glucagon-like peptide-1 receptor agonists and other diabetes therapies: a secondary analysis of the MAESTRO-NASH trial. Aliment Pharmacol Ther. (2025) 62(11-12):1089–99. 10.1111/apt.70382 41127972 PMC12646629

[165] James S Erlinge D Storey RF McGuire DK De Belder M Eriksson N. Dapagliflozin in myocardial infarction without diabetes or heart failure. NEJM Evidence. (2024) 3(2):EVIDoa2300286. 10.1056/EVIDoa2300286 38320489

[166] Guirguis E Dougherty J Thornby K Grace Y Mack K. Resmetirom: the first food and drug administration-approved medication for nonalcoholic steatohepatitis (NASH). Ann Pharmacother. (2025) 59(2):162–73. 10.1177/10600280241259528 38887011

[167] Lin Y Song J Li X. Identification of biomarkers between coronary artery disease and non-alcoholic steatohepatitis: a combination of bioinformatics and machine learning. Front Genet. (2025) 16:1573621. 10.3389/fgene.2025.1573621 40747105 PMC12310482

[168] Di H Wang S Xu C Yin Q Xu K Zheng W. Shared genetic links between nonalcoholic fatty liver disease and coronary artery disease. Glob Heart. (2024) 19(1):88. 10.5334/gh.1374 39619635 PMC11606392

[169] Xu T Gao W Zhu L Chen W Niu C Yin W. NAFLDkb: a knowledge base and platform for drug development against nonalcoholic fatty liver disease. J Chem Inf Model. (2024) 64(7):2817–28. 10.1021/acs.jcim.3c00395 37167092

[170] Altaf-Ul-Amin M Nasution AK Islam RM Gao P Ono N Kanaya S. Drug repurposing for non-alcoholic fatty liver disease by analyzing networks among drugs, diseases, and genes. Metabolites. (2025) 15(4):255. 10.3390/metabo15040255 40278384 PMC12029302

[171] Lin A Wang Z Jiang A Chen L Qi C Zhu L. Large language models in clinical trials: applications, technical advances, and future directions. BMC Med. (2025) 23(1):563. 10.1186/s12916-025-04348-9 41088200 PMC12522288

[172] Rinella ME Neuschwander-Tetri BA Siddiqui MS Abdelmalek MF Caldwell S Barb D. AASLD Practice guidance on the clinical assessment and management of nonalcoholic fatty liver disease. Hepatology. (2023) 77(5):1797–835. 10.1097/HEP.0000000000000323 36727674 PMC10735173

[173] Vilar-Gomez E Martinez-Perez Y Calzadilla-Bertot L Torres-Gonzalez A Gra-Oramas B Gonzalez-Fabian L. Weight loss through lifestyle modification significantly reduces features of nonalcoholic steatohepatitis. Gastroenterology. (2015) 149(2):367–378.e5. 10.1053/j.gastro.2015.04.005 25865049

